# The glucocorticoid receptor is affected by its target ZBTB16 in a dissociated manner

**DOI:** 10.1530/JOE-24-0283

**Published:** 2025-07-04

**Authors:** Sekar Galuh, Erin Faught, Ingeborg Klaassen, Lisa L Koorneef, Joost Brinks, Elon H C van Dijk, Dirk Elewaut, Reinier O Schlingemann, Marcel J M Schaaf, Camiel J F Boon, Onno C Meijer

**Affiliations:** ^1^ Department of Ophthalmology, Leiden University Medical Center, Leiden, The Netherlands; ^2^ Institute of Biology Leiden, Leiden University, Leiden, The Netherlands; ^3^ Ocular Angiogenesis Group, Department of Ophthalmology, Amsterdam University Medical Center, Amsterdam, The Netherlands; ^4^ Molecular Immunology and Inflammation Unit, VIB-UGent Center for Inflammation Research, Zwijnaarde, Belgium; ^5^ Department of Anatomy and Embryology, Leiden University Medical Center, Leiden, The Netherlands; ^6^ Department of Internal Medicine and Pediatrics, Faculty of Medicine and Health Sciences, Ghent University, Ghent, Belgium; ^7^ Department of Rheumatology, Ghent University Hospital, Ghent, Belgium; ^8^ Department of Ophthalmology, Amsterdam University Medical Center, Amsterdam, The Netherlands; ^9^ Department of Ophthalmology, University of Lausanne, Jules Gonin Eye Hospital, Fondation Aisle Des Aveugles, Lausanne, Switzerland; ^10^ Department of Medicine, Division of Endocrinology and Metabolism, Leiden University Medical Center, Leiden, The Netherlands

**Keywords:** cortisol, PLZF, transactivation, transrepression, intracellular feedback

## Abstract

The glucocorticoid receptor (GR) mediates many activating and repressive effects of glucocorticoids in multiple contexts. Glucocorticoids can robustly induce the transcriptionally active protein Zinc finger and BTB domain containing 16 (ZBTB16). We evaluated how cortisol-induced ZBTB16, in turn, affects various GR-mediated actions in human cells and in zebrafish. We found that prevention of *ZBTB16* induction led to potentiated GR-dependent effects on the human endothelial cell barrier and blood glucose levels in zebrafish larvae. In contrast, *zbtb16* functional knockout abolished the GR-dependent effects on the inflammatory response in zebrafish larvae. At the mRNA level, *zbtb16* knockdown potentiated transactivation and attenuated transrepression in a subset of GR target genes. Finally, ZBTB16 protein was strongly induced by dexamethasone in fibroblast-like synoviocytes derived from osteoarthritis patients. The data suggest that cortisol-induced ZBTB16 acts as an intracellular modulator of glucocorticoid action by limiting GR-mediated activating effects and enhancing repressive effects. This mechanism may facilitate a return to the initial cellular state after (proinflammatory) stimulation and enhance GR’s anti-inflammatory effects. This mechanism is similar to that of ‘dissociated’ GR ligands and may guide drug development that aims to reduce side effects while retaining the clinical benefits of glucocorticoid treatment.

## Introduction

Glucocorticoid hormones are secreted by the adrenal cortex in a circadian pattern and in response to stressors under control of the hypothalamus–pituitary–adrenal (HPA) axis to maintain or restore homeostasis (Timmermans *et al.* 2019). Cortisol is the main glucocorticoid in humans. Many stress-related effects (either acute or chronic) of glucocorticoids are mediated via the glucocorticoid receptor (GR). These effects include eliciting metabolic changes, initiating negative feedback on the HPA axis, and mediating anti-inflammatory responses in the context of infection and autoimmunity (Sapolsky *et al.* 2000, Coutinho & Chapman 2011). The GR is expressed in all tissues and many cell types. Upon binding of cortisol, the GR translocates to the cell nucleus to regulate the expression of its target genes. For most of the induced target genes, the GR binds to palindromic glucocorticoid responsive elements (GREs), followed by recruitment of co-activators that mediate transcriptional activation (‘transactivation’). Repression of target genes may involve binding to negative GREs (nGREs), prompting co-repressor recruitment to establish transcriptional repression. Alternatively, GRs interact with other transcription factors to (mostly) repress their activity (Oakley & Cidlowski 2013), which provides a mechanism for the restoration of homeostasis after stressors, such as immune challenges (Sapolsky *et al.* 2000).

GR activation is subject to intracellular modulation via its own target genes. GR suppresses transcription of the *NR3C1* gene, which encodes the GR protein itself, in a process of homologous downregulation (Oakley & Cidlowski 1993). A second intracellular feedback mechanism is the GR-mediated induction of the *FKBP5* gene. The FKBP51 protein acts as a chaperone of cytoplasmic GR and limits its activation by cortisol and transcriptional activation (as it does with other steroid receptors like the progesterone receptor (Hubler *et al.* 2003, Wochnik *et al.* 2005)). The physiological necessity to limit GR activation is clear from many detrimental effects that come with overexposure to glucocorticoids, which may, for example, be associated with Cushing’s disease, side effects from synthetic GC use, or chronic stress (Ramamoorthy & Cidlowski 2013, Spies *et al.* 2021).

In the context of our recent studies in cortisol-induced disease using primary choroidal endothelial cells (CECs) derived from human eyes, zinc finger and BTB domain containing 16 (*ZBTB16*) emerged as the most pronounced GR target gene (Brinks *et al.* 2022). *ZBTB16*, or *PLZF*, encodes a transcription factor that belongs to the POZ-Krüppel family. Most cell types express *ZBTB16* at very low levels in unstimulated conditions, but a robust induction of *ZBTB16* expression by glucocorticoids has been reported in numerous cell lines, such as human endometrial cells (Fahnenstich *et al.* 2003, Arlier *et al.* 2023), natural killer T (NKT) cells (Mao *et al.* 2016), leukemia cell lines (Wasim *et al.* 2010), human pancreatic islets (Aylward *et al.* 2021), breast cancer cell lines (Prekovic *et al.* 2023), and cerebral organoids (Krontira *et al.* 2024). However, the overall role of ZBTB16 as an effector or modulator of GR-mediated processes is poorly understood.

In the current study, we evaluated the expression and effects of glucocorticoid-induced *ZBTB16* in three different contexts. In human primary CECs, our original cell type of interest, we observed that ZBTB16 exerted intracellular feedback on GR activity. We extended this notion in zebrafish larvae, based on their clear advantages in terms of manipulation of gene expression and the possibility to study a wider range of functional effects. Finally, GR-mediated induction of ZBTB16 protein in synovial fibroblasts from rheumatoid arthritis patients suggested that ZBTB16 induction can play a role in a clinically relevant cell type. Our data suggest that cortisol-induced *ZBTB16* expression limits specific effects of cortisol through attenuation of GR-mediated transactivation, while it potentiates GR-mediated transrepression. This mechanism suggests a newly identified intracellular autoregulatory mechanism of GR activation that is conserved across species.

## Materials and methods

### Ethics statement

All experiments were performed in accordance with the relevant ethical guidelines. The study protocol for post-mortem eye donors was approved by the local ethics committee of LUMC and followed the Declaration of Helsinki on the use of human material for research. The donors gave written informed consent for organ and tissue donation, in accordance with Dutch laws. The study protocol involving osteoarthritis patients was approved by the Ghent University Hospital Ethics Committee (BC-06496). Informed consent was obtained from all participating patients. The human serum used in the siRNA-mediated knockdown of *ZBTB16* in primary CECs was approved by the Medical Ethical Review Committee of the Academic Medical Center Amsterdam. Subjects gave informed consent for the use of serum and samples were stored anonymously. Human serum was collected according to the principles of conduct for research integrity in the Amsterdam UMC Research Code (https://www.amsterdamumc.org/en/research/research-code.htm).

### Isolation and culture of human primary choroidal endothelial cells

Eyes from a total of six human donors, which were enucleated within 24 h postmortem, were obtained from the Department of Anatomy and Embryology at Leiden University Medical Center (LUMC) or the Netherlands Brain Bank (Table 1). The donors' medical histories, including ocular and vascular diseases, were not known. Donors were mostly female (5 out of 6), but previously we did not observe any sex-specific effects on cortisol responsiveness in cultured primary CECs (Brinks *et al.* 2022).

**Table 1:** Origin of human primary CECs.

Donor #	Sex	Age (years)	Cause of death
Donor 1	Female	98	Unknown
Donor 2	Female	88	Unknown
Donor 3	Male	80	Unknown
Donor 4	Female	98	Terminal phase of Alzheimer disease
Donor 5	Female	70	End-stage frontotemporal dementia
Donor 6	Female	72	Euthanasia

CECs, choroidal endothelial cells.

The isolation protocol of human primary CECs was performed as previously described with minor modifications (Brinks *et al.* 2018). In brief, the enucleated eyes were washed briefly with 70% ethanol and cold phosphate-buffered saline (PBS) containing 1% penicillin-streptomycin (15140122, Gibco Life Technologies, USA). In a petri dish, extraocular tissue, anterior part of the eye, and vitreous humor were removed, thus leaving the posterior pole of the eye. After removing the neuroretinal tissue layer, the complex of retinal pigment epithelium (RPE) and choroid layer was carefully detached from sclera, incubated with 0.05% Trypsin–EDTA for 1 h at 37°C in a shaking water bath, and transferred to PBS. The excess RPE was gently brushed using a camel hair brush under a dissection microscope (MZ6; Leica, Germany). The tissue was then cut into small pieces, incubated with 0.2% collagenase (type II) (LS004176, Worthington Biochem, USA) for 1.5 h at 37°C in a shaking water bath. The digestion process was stopped by adding Dulbecco’s modified Eagle’s medium (DMEM) without pyruvate (Gibco Life Technologies) supplemented with 10% fetal calf serum (FCS) (Sigma Aldrich, USA). The cell suspension was filtered using MACS® SmartStrainers 30 μm (130-098-458, Miltenyi Biotec, Germany) and centrifuged at 232 ***g*** for 5 min. The primary CECs were isolated by incubating the cells with Dynabeads^TM^ CD31 (11155D, Thermo Fisher Scientific) and processed with DynaMag^TM^-15 Magnet (12301D, Thermo Fisher Scientific) according to the manufacturer’s instruction. The purity of CECs was confirmed with flow cytometry detecting endothelial cell markers (CD31 (PECAM-1) WM-59 (14-0319-82, Invitrogen, USA) and CD144 (VE-Cadherin) 16B1 (12-1449-82, Invitrogen)) that reached purity between 80 and 90%, shown in Supplementary Fig. S1A, B, C, D, E (see section on Supplementary materials given at the end of the article). Fibroblast growth was monitored during cell culture. The expression of endothelial (*VWF* and *CD34*) gene markers was confirmed in the isolated primary CECs. The expression of EC markers was highly similar across donors, with the exception of donor 3, which suggests the presence of fibroblasts in the culture of this donor (Supplementary Fig. S1F and G).

Primary CECs were cultured with EGM-2 MV medium that consists of EBM^TM^-2 basal medium (CC-3156; Lonza, USA) and EGM^TM^-2 MV SingleQuots^TM^ Supplement pack (CC-4147; Lonza) without hydrocortisone supplement but containing 5% fetal bovine serum (FBS), and maintained in an incubator with 5% CO_2_ at 37°C. The cells were used for experiments when confluent, until passage 7.

### Impedance for barrier integrity measurement

Primary CECs were seeded at 40,000 cells/well onto a fibronectin-coated thinCert® (662640; Greiner Bio-One, Switzerland) in EGM-2 MV medium (including 5% FBS, but without hydrocortisone supplement). The inserts were transferred to an automated cell monitoring system (CellZscope®, NanoAnalytics, Germany), which was placed inside an incubator with 5% CO_2_ at 37°C, with the external controller connected to a computer running the CellZscope® software (version 4.4). Three days after seeding, the cells were treated with vehicle (0.01% ethanol), 3 nM cortisol (hydrocortisone; H4001, Sigma-Aldrich), or 100 nM cortisol for 24 h, in some cases co-incubated with 1,000 nM of the GR antagonists mifepristone (RU-486) or relacorilant (both gifts from Corcept Therapeutics, USA). The transendothelial electrical resistance (TEER) and capacitance (C_cl_) of the cells were recorded every 15 min, with the reference insert automatically subtracted from the output value each time in the same run. The TEER value was considered valid when C_cl_ was in the range between 0.5 and 5.0 μF/cm^2^.

### Small interfering RNA-mediated knockdown of *ZBTB16* in primary CECs

Silencing *ZBTB16* in cultured CECs was performed by treating the cells with ON-TARGETplus SMARTpool mix small interfering RNA (siRNA) (Dharmacon, UK) from Horizon Discovery,UK. We used a reverse transfection method, in which the transfection mixtures were added onto the coated wells before cell seeding, per the manufacturer’s instruction and previously validated (Bosma *et al.* 2023). Briefly, primary CECs were treated with 25 nM either mix siRNA *ZBTB16* (si*ZBTB16*; L-018719-00-0005) or non-targeting control mix siRNA (siNT; D-001810-10-05), and 2.5 μg/mL DharmaFECT^TM^ transfection reagent 1 (Dharmacon) in M-199 medium supplemented with 2% heat-inactivated human serum. The medium was replaced with primary CEC medium 6 h after transfection to avoid cytotoxicity. Pharmacological treatment (vehicle, 100 nM cortisol, or 1,000 nM cortisol) was applied 48 h after transfection for 4 h to determine gene expression, or 24 h to determine protein expression and evaluate barrier integrity.

### Immunofluorescence

Primary CECs were seeded at 25,000 cells per well on fibronectin-precoated coverslips 13 mm^2^. At near confluency, the cells were treated with vehicle (0.01% ethanol) or 100 nM cortisol for 24 h, followed by fixation with 4% paraformaldehyde in Dulbecco’s phosphate-buffered saline (DPBS) for 15 min. We used DPBS containing Mg^2+^ and Ca^2+^ (14040133, Thermo Fisher Scientific) for all washing steps. Next, the cells were incubated with blocking solution containing 2% BSA, 0.3% Triton X-100, and DPBS for 1 h at room temperature (RT). Afterward, the cells were incubated overnight at 4°C with primary antibody against ZBTB16 (1:50, MA5, Sigma-Aldrich) in DPBS and 0.3% Triton X-100, followed by two DPBS washes, each for 10 min. The cells were then incubated for 2 h at RT with secondary antibody donkey anti-mouse (Alexa Fluor^TM^-594, A21203, Invitrogen, Thermo Fisher Scientific) at 1:1,000 dilution and washed with DPBS two times for 10 min each. Finally, the coverslips were mounted with ProLong^TM^ Gold Antifade containing 4′,6-diamidino-2-phenylindole (DAPI) (P36931, Thermo Fisher Scientific), which also stained the nuclei.

Immunofluorescence images were acquired with a Leica SP8 confocal laser scanning microscope (Leica, Germany) through an oil immersion lens of HC PL FLUOTAR 100×/1.30. The laser lines were set at 405 and 594 nM and detected with HyD detector (410–483 nM and 586–751 nM, respectively). Line sequential scanning was chosen to avoid signal crosstalk. All images were processed with LAS X software (version 3.5.7.23225 and version 3.7.1.21655). The red channel was turned to green in the visualization to provide more contrast with the nuclei.

### Culture of primary human umbilical vein endothelial cells

A pooled primary human umbilical vein endothelial cells (HUVECs) from five different donors was obtained from the Department of Vascular Surgery at LUMC. HUVECs were cultured in a fibronectin-precoated wells and maintained in EGM-Plus medium (CC-5035; Lonza) without hydrocortisone supplement, but containing 2% FBS, and maintained in an incubator with 5% CO_2_ at 37°C. The cells were used maximally at passage 9. The procedure for silencing *ZBTB16* mRNA in HUVECs was similar to that used for primary CECs, except that HUVEC culture medium was used for maintenance.

For PLA, HUVECs were seeded at 80,000 cells per well on precoated coverslips 13 mm^2^. The next day, the cells were treated with vehicle (0.01% ethanol), 100 nM cortisol, or 100 nM dexamethasone (D1756, Sigma-Aldrich) for 24 h. Then, the cells were fixed with 4% paraformaldehyde for 15 min at RT.

### Duolink® proximity ligation assay

The *in situ* PLA was performed using Duolink® PLA reagents (Sigma-Aldrich) according to the manufacturer’s instructions. All incubations throughout the assay were performed in a humid condition at 37°C. Before starting the experiment, the fixed HUVECs on coverslips were permeabilized with 0.3% Triton X-100 for 10 min at RT. Then, the cells were incubated with Duolink® blocking solution buffer for 1 h. Primary antibodies against GR (D6H2L, Cell Signaling Technology, The Netherlands), Lamin A/C (4C11, Cell Signaling Technology), and ZBTB16 (MA5, Thermo Fisher Scientific) were diluted 1:50, 1:100, and 1:50 (respectively) in Duolink® Antibody Diluent. The cells were incubated with primary antibodies for overnight at 4°C. After washing the primary antibodies with Wash Buffer A two times for 5 min each, the primary antibodies were probed using Duolink® *in situ* PLA probes rabbit PLUS (1:5, DUO92002, Sigma-Aldrich) and mouse MINUS (1:5, DUO92004, Sigma-Aldrich) in Duolink® Antibody Diluent for 1 h, followed by two 5 min washes with Wash Buffer A. Subsequently, cells were incubated with ligase (1:40) in diluted Duolink® Ligation buffer (1:5) for 30 min. After two washes of 5 min each, the cells were incubated with polymerase (1:80) in diluted amplification buffer (1:5) for 100 min. The cells were protected from light from this point onward. At the final wash step, the cells were washed two times with Wash Buffer B and one time with 0.01× Wash Buffer B. Finally, the coverslips were mounted using ProLong^TM^ Gold Antifade containing DAPI (P36931, Thermo Fisher Scientific).

The PLA images were processed as mentioned in the immunofluorescence protocol. The images were acquired through an oil immersion lens of HC PL APO CS2 63×/1.40. The laser lines were set at 405 and 594 nM and detected with HyD detector (410–547 nM) and PMT detector (591–752 nM). All images were processed with LAS X software (version 3.5.7.23225 and version 3.7.1.21655).

### Zebrafish larvae maintenance

The adult zebrafish were maintained as described previously (van Mever *et al.* 2024). Briefly, adult zebrafish were maintained in a recirculating system (Fleuren & Nooijen, The Netherlands) under a 14 h light:10 h darkness cycle. Water was maintained at 28.5°C, pH 7.4, 300 μS conductivity, and 10% of the water was exchanged daily. Animals were fed twice daily with Gemma Micro 500 diet (Skretting, USA) in the morning and live *Artemia* (Great Salt Lake Brine Shrimp Cooperative, USA) in the afternoon. Zebrafish embryos/larvae were maintained in a 28.5°C incubator in 10 × 100 mm^2^ Petri dishes in E3 medium with daily water changes, according to the guidelines from the zebrafish Model Organism Database (https://zfin.org/). The zebrafish lines used in this study were either the wild-type (WT) strain AB/TL or, to study the innate immune response in two cell lineages, a double transgenic line *Tg (mpx:GFP/mpeg1:mcherry-F)*, with fluorescently tagged macrophages (mpeg:mCherry) and neutrophils (mpx:eGFP) (Renshaw *et al.* 2006, Bernut *et al.* 2014).

### Functional knockout of *zbtb16* in zebrafish F0 mutants

Zebrafish embryos were microinjected with Cas9 protein/synthetic crRNA:tracrRNA duplex (RNPs) (hereafter called crispants) at the single-cell stage before cell inflation (Kroll *et al.* 2021). The Alt-R® CRISPR-Cas9 system from Integrated DNA Technologies was used. Briefly, synthetic crRNA-specific sites targeting *zbtb16a* and *zbtb16b* (Alt-R® CRISPR-Cas9 crRNA, 2 nmol) were designed using CHOPCHOP ver.3 (Labun *et al.* 2019) (Supplementary Table S1). One pair of gRNAs, each targeting *zbtb16a* (crRNA Target 2) and *zbtb16b* (crRNA Target 2), was prepared according to the protocol by the annulation of each crRNA to tracrRNA (1072533; Alt-R® CRISPR-Cas9 tracrRNA). At the single-cell stage and before cell inflation, zebrafish embryos were injected with pooled RNP (pre-assembled Cas9 protein (1081059; Alt-R® S.p. Cas9 Nuclease V3) and gRNA with 1-to-1 ratio). All materials were ordered from Integrated DNA Technologies unless otherwise stated. To identify potential insertion/deletions (indels), we genotyped by fragment analysis and visualized shifts in amplicon size by quantitative PCR using SYBR-generated melt curves.

To determine whether a loss of *zbtb16a/b* impacted GR transcriptional activation, larvae at 4 days post-fertilization (dpf) were treated with 5 μg/mL cortisol/hydrocortisone (Sigma-Aldrich) or vehicle (0.05% ethanol) for 20 h. Larvae were euthanized with an overdose of MS-222 (0.4 g/L) and stored at −80°C until further analysis.

### Tail fin amputation and image quantification

Resection of the tail fin fold (tail fin amputation) was performed on WT larval zebrafish or crispants generated in the double transgenic line *Tg (mpx:GFP/mpeg1:mcherry-F)*. To determine whether a loss of *zbtb16a/b* impacted GR-mediated transrepression, larvae were treated with cortisol as described above. After 20 h of cortisol or vehicle treatment, tail fin amputation was performed as described previously (Xie *et al.* 2019).

The number of leukocytes that migrated toward the wound (within 200 μm) was imaged using a Leica M205FA fluorescent stereomicroscope with a Leica DFC 345 FX camera and quantified by manual counting. Macrophages were detected based on their mCherry fluorescence tag, while neutrophils were distinguished by the GFP fluorescence tag.

### Glucose measurement

Glucose levels were determined enzymatically (Faught & Vijayan 2019), using β-D-glucose as a standard. Briefly, the assay is based on the generation of reduced nicotinamide adenine dinucleotide (NADH; abs 340 nm), which results from the conversion of glucose to 6-phosphogluconate. The assay was run using whole-body lysates of zebrafish larvae, where the lysate was generated from a pool of ten larvae homogenized in 50 mM Tris, pH 7.5.

### RNA extraction and quantitative PCR

To determine the transcript abundance of the target genes, we performed quantitative PCR (qPCR) as previously described (Brinks *et al.* 2022, Faught & Schaaf 2023). Total RNA from primary CECs was isolated using TriPure^TM^ (Roche, Switzerland) and quantified using a Nanodrop spectrophotometer. Complementary DNA (cDNA) was synthesized using M-MLV reverse transcriptase and RNasin® (N2111; Promega, USA). For zebrafish larvae, total RNA was extracted from pools of ten larvae using TRIzol reagent (Thermo Fisher Scientific), according to the manufacturer’s instructions. One μg RNA was treated with RNase-free DNase I (EN0521; Thermo Fisher Scientific) to remove genomic DNA contamination. The High-Capacity cDNA Reverse Transcription Kit (4368814; Applied Biosystems, The Netherlands) was used to synthesize cDNA, following the manufacturer’s protocol. The qPCR was performed using SYBR Green master mix (Promega) and run with Bio-Rad CFX Maestro software version 2.2 (Bio-Rad Laboratories, USA). Gene-specific primer sequences are listed in Supplementary Tables S2 and S3. In all cases (genotyping and transcript data), melt curves were run, and the presence of a single amplification product was confirmed.

### Western blot

The subcellular protein fractions of primary CECs were extracted using NE-PER^TM^ Nuclear and Cytoplasmic Extraction Reagents (78833; Thermo Fisher Scientific), according to the manufacturer’s instructions. Protein concentration was determined using Pierce^TM^ BCA protein assay kits (23225; Thermo Scientific). Subnuclear fraction (9 ng) proteins were loaded, separated on a pre-cast 10% polyacrylamide gel (456-8035; Bio-Rad Laboratories), and blotted to a Trans-Blot Turbo Midi 0.2 μm nitrocellulose membrane (1704159; Bio-Rad Laboratories).

The membrane was blocked for 1 h at RT, incubated overnight at 4°C with the primary antibodies, and incubated for 90 min at RT with secondary antibodies (Supplementary Table S4). Chemiluminescence of protein bands was detected using Pierce^TM^ ECL Substrate (Thermo Fisher Scientific) and visualized using ChemiDoc^TM^ Touch Imaging System (Bio-Rad Laboratories). The protein bands were analyzed and quantified with Image Lab software version 6.1.0 (Bio-Rad Laboratories).

### Isolation and culturing of human fibroblast-like synoviocytes (FLS)

We validated the protein abundance of ZBTB16 in patients with osteoarthritis using mass spectrometry-based proteomics. These samples were part of a study at the VIB Institute and Ghent University, of which the other results will be published elsewhere. Synovial tissues were obtained during joint replacement surgery from four female osteoarthritis patients with a BMI >25, who were not using GR agonists. FLS were obtained by enzymatic digestion as previously described (Müller-Ladner *et al.* 1996) and grown in DMEM (Invitrogen) supplemented with 10% FCS (Invitrogen). FLS were used at passages 4–8 for experiments in three technical replicates and serum-starved for 16 h before the experiment. Cells were pre-incubated with dexamethasone (0.005 μM, D4902; Sigma-Aldrich) or vehicle (0.05% DMSO) for 1 hour, after which human tumor necrosis factor-alpha (TNF-α) (10 ng/mL, in-house production, VIB Protein Core) was added for an additional 23 h. Cells were washed with ice-cold PBS, collected with 5% sodium dodecyl sulfate (SDS)/50 mM triethylammonium bicarbonate (TEAB), snap-frozen, and stored at −80°C until further processing. The liquid chromatography with tandem mass spectrometry (LC-MS/MS) analysis procedure can be found in the Supplementary Information.

### Statistical analysis

Before fold change calculation (Fig. 2), the ΔCT value was converted to an absolute value, then corrected for interindividual variation with Factor Correction software (version 10.5), as described (Ruijter *et al.* 2006). Relative gene expression (Fig. 4) was calculated using ΔΔCT method, and ΔCT values were used for statistical analysis. The normality of each dataset was tested using the Shapiro–Wilk test. All statistical analyses, except for proteomics data, were performed using a paired *t*-test, an unpaired *t*-test, or a 2-way ANOVA followed by Tukey’s multiple comparison test in GraphPad Prism (version 10.2.3 for Windows; GraphPad Software, USA). A *P*-value <0.05 was considered statistically significant.

For the proteomics data, the data were log2-normalized, and missing protein intensity values were imputed by randomly sampling from a normal distribution centered around each sample’s noise level using the *DEP* package (Zhang *et al.* 2018). The comparison between sample group means was performed using the *limma* package (Ritchie *et al.* 2015), with a false discovery rate <0.05, a log fold change of >2- or <0.5-fold (|log2FC| = 1), and an adjusted *P*-value <0.05.

## Results

### GR-induced ZBTB16 limits the functional barrier of human primary CECs

**Figure 1:**
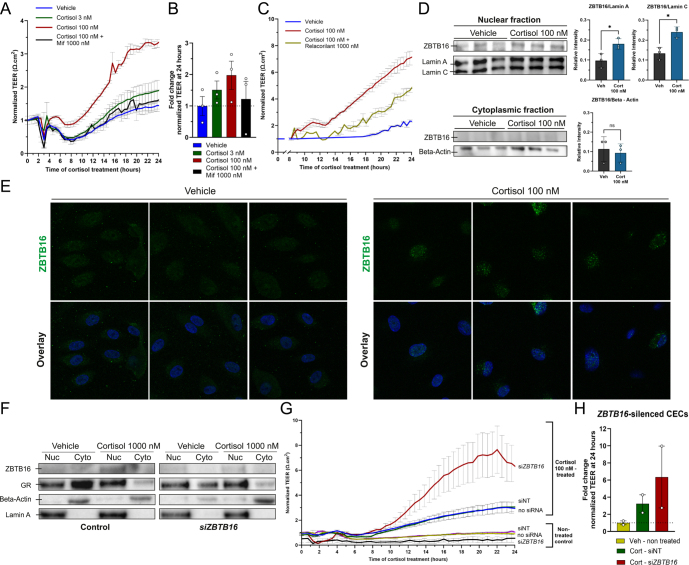
Cortisol, via GR, enhances endothelial barrier integrity, and this effect is exaggerated after *ZBTB16* silencing. (A) Cortisol dose-dependently increased the barrier integrity of primary CECs measured by TEER, and this was blocked by mifepristone (Mif) (*n* = 3 individual donors). The spectra were normalized to the TEER value 1 h before the treatment (mean ± S.E.M). (B) The fold change of cortisol effect on average TEER value across group of treatments (mean ± S.E.M). (C) Co-incubation with 1,000 nM of the selective GR antagonist, relacorilant, attenuated endothelial barrier integrity induced by 100 nM cortisol (mean ± S.D). (D) After 24 h, 100 nM cortisol (Cort) induced ZBTB16 protein expression in the nuclear fraction compared to the vehicle treatment (mean ± S.D). (E) Representative images of immunofluorescence staining of primary CECs (donor 6) that were treated with vehicle or 100 nM cortisol for 24 h. ZBTB16 (green) was present in the DAPI-stained nuclei (blue) after cortisol treatment. (F) Induction of ZBTB16 protein expression after 24 h of 1,000 nM cortisol treatment in the nuclear (Nuc) fraction was absent in *ZBTB16*-silenced CECs (48 h post-transfection). (G) In the *ZBTB16*-silenced CECs, the cortisol-induced increase in barrier integrity (normalized TEER value) was exaggerated after 24 h of treatment compared to the WT CECs (*n* = 2 individual donors, mean ± S.E.M). (H) The fold change of cortisol effect on average TEER value from *ZBTB16*-silenced CECs (mean ± S.E.M). Each experiment was performed in 3–4 technical replications. siNT: non-target control siRNA, siRNA: small interfering RNA, si*ZBTB16*: siRNA *ZBTB16*, veh: vehicle.

In the context of the effects of cortisol on barrier function and permeability of the choroidal endothelium (Brinks *et al.* 2021), we studied how cortisol affects the functional properties of primary CECs. We measured the effect of cortisol on the barrier integrity of isolated primary CECs from three donors. Our findings showed that cortisol promoted a dose-dependent increase of TEER values with ∼1.2-fold increase by low dose of cortisol and ∼two-fold by high dose of cortisol (Fig. 1A and B). This regulation was mediated by GR, as co-incubation with both the non-selective GR antagonist mifepristone and the GR-selective antagonist relacorilant (Viho *et al.* 2022) prevented the effect (Fig. 1A and C).

Our previous work showed that cortisol dose-dependently upregulated the *ZBTB16* expression via GR in human endothelial cells (Brinks *et al.* 2022). In line with this, ZBTB16 protein was lowly expressed under unstimulated conditions, but was induced by cortisol in the nuclear fraction after 24 h of stimulation (Fig. 1D and E), conforming to our previous findings (Brinks *et al.* 2022). Initially, we hypothesized that GR-induced ZBTB16 mediated cortisol-dependent barrier strengthening. To test this, we silenced the *ZBTB16* mRNA using siRNA and simultaneously monitored CECs’ barrier integrity in real time. *ZBTB16* knockdown was validated 24 h after treatment (72 h post-transfection) in the nuclear fraction of cortisol-treated cells (Fig. 1F). We observed a robust increase in barrier function upon cortisol treatment, and this effect was exaggerated upon knockdown of *ZBTB16* by ∼6.4 fold (Fig. 1G and H). This suggests that ZBTB16 induction by GR activation is not a mediator of the effect of cortisol on endothelial barrier integrity, but rather limits the magnitude of the cortisol effect on the barrier.

### Cortisol-induced ZBTB16 modulates the GR-mediated transactivation and transrepression in a gene-specific manner

**Figure 2:**
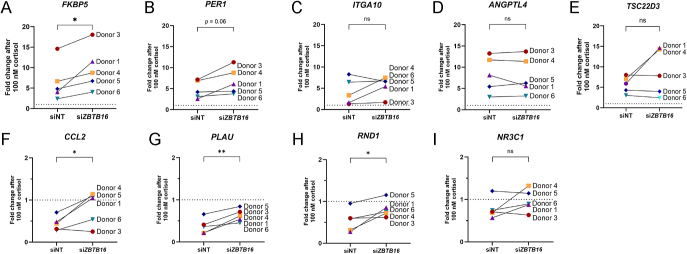
*ZBTB16* silencing in primary CECs affects GR-mediated gene expression. Primary CECs derived from five post-mortem eye donors were transfected with either SMARTpool mix si*ZBTB16* or siNT for 48 h. Cortisol-induced GR target genes were measured after treatment with 100 nM cortisol (Cort) for 4 h (upper row): (A) *FKBP5* (*P* = 0.04), (B) *PER1*, (C) *ITGA10*, (D) *ANGPTL4*, (E) *TSC22D3* (*GILZ*). The lower row shows repressed genes: (F) *CCL2* (*P* = 0.048), (G) *PLAU* (*P* = 0.009), (H) *RND1* (*P* = 0.047), (I) *NR3C1*. The Ct value of target gene was normalized to *LRP10* as a housekeeping gene. The black lines connecting two points show the change between siNT and siZBTB16 conditions within the same donor. ns: not significant.

We postulated that the limiting effect of ZBTB16 on GR activity would also be apparent at the level of gene expression. To investigate this, we treated primary CECs from five post-mortem eye donors for 48 h with si*ZBTB16* or siNT. After subsequent 4 h of stimulation with 100 nM cortisol, we evaluated expression of previously established GR-induced target genes in these cells (Brinks *et al.* 2022). Our results showed that cortisol increased the *FKBP5* mRNA levels under non-targeted control conditions, and this increase was exaggerated ∼1.5-fold in the *ZBTB16*-silenced cells (Fig. 2A and Supplementary Fig. S2A). Cortisol also induced *PER1*, *ITGA10*, *ANGPTL4*, and *TSC22D3* (*GILZ*) mRNA expression in the non-targeted, but this induction was not statistically different in the silenced cells (Fig. 2B, C, D, E and Supplementary Fig. S2B, C, D, E). This suggests that ZBTB16 induction by GR activation suppressed transactivation-dependent genes in a gene-specific manner.

We also evaluated four genes of which the expression is known to be repressed via GR (Fig. 2F, G, H, I). Downregulation of *CCL2, PLAU*, and *RND1* mRNA was significantly attenuated upon knockdown of *ZBTB16* (Fig. 2F, G, H and Supplementary Fig. S2F, G, H, respectively). In contrast, downregulation of *NR3C1* expression was not attenuated in the *ZBTB16*-silenced cells (Fig. 2I and Supplementary Fig. S2I). Interindividual donor variation was inevitably observed for both the silencing efficiency (Supplementary Fig. S3A) and target genes. However, there was no significant correlation between the silencing efficiency and the ratio of cortisol induction with si*ZBTB16* versus siNT (Supplementary Fig. S3B). Overall, the gene expression data from cultured primary CECs indicate that GR-mediated transactivation is attenuated by ZBTB16, while it contributes to transrepression, both aspects showing gene specificity.

### Modest PLA signal for dexamethasone-, but not cortisol-induced ZBTB16 and GR

The gene-specific effect by ZBTB16 modulation may involve ZBTB16 binding to the GR complex. To investigate a possible interaction between ZBTB16 and GR in the cell nucleus, we performed PLA that allows us to identify *in situ* protein–protein proximity at distances less than 40 nm. We used primary HUVECs in this experiment due to limited availability of primary CECs. We confirmed the induction of ZBTB16 gene and protein expression in HUVECs after glucocorticoid treatment (Supplementary Fig. S4A, B, C). In line with primary CECs, GR-mediated transactivation upon cortisol was exaggerated (Supplementary Fig. S5A, B, C, D, E), and GR-mediated gene repression was attenuated in *ZBTB16*-silenced HUVECs (Supplementary Fig. S5F, G, H, I). These data confirm the relevance of HUVECs in investigating the interaction between ZBTB16 and GR.

**Figure 3:**
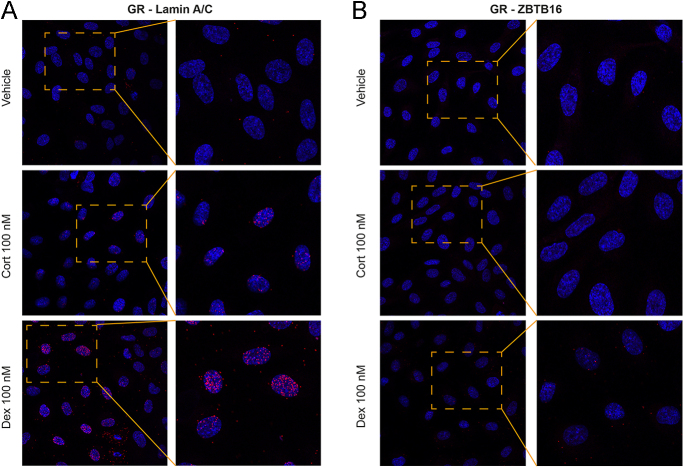
Minimal detection of GR and ZBTB16 close proximity. Primary HUVECs pooled from five donors were treated with either vehicle, 100 nM cortisol (Cort), or 100 nM dexamethasone (Dex). PLA was performed after the cells were probed with primary antibodies. (A) As positive control, Cort and Dex increased the PLA signal (red dots) for GR and Lamin A/C in the nuclei. (B) In the cells probed with antibodies against GR and ZBTB16, Dex but not Cort showed a very modest PLA signal.

A recent study described a corticosterone-induced PLA signal between GR and Lamin A/C at the inner nuclear membrane and extending into the nucleoplasm (Paul *et al.* 2025). In line with this finding, cortisol and dexamethasone induced GR-Lamin A/C interactions, shown by red dots (PLA signal), in the nuclei of ECs (Fig. 3A). This signal served as a positive control for our experiments. In contrast, no PLA signal was observed for GR and ZBTB16 with 100 nM cortisol. In the 100 nM dexamethasone condition, there was a very low but consistent PLA signal (Fig. 3B). This suggests that GR and ZBTB16 may be in close proximity or in the same complex, but that this is detectable only with maximal GR activation.

### Cortisol-induced expression of *zbtb16* and modulation of GR-mediated gene transcription by Zbtb16 is conserved in zebrafish larvae

To further investigate the biological effects of *zbtb16* induction by cortisol in a functional *in vivo* context, we used zebrafish larvae as a model because of their genetic traceability and well-characterized stress response (Chatzopoulou *et al.* 2016), as well as its versatility in genetic manipulation (Chatzopoulou *et al.* 2015). Previous work established that zebrafish have two paralogs of *zbtb16* (*zbtb16a* and *zbtb16b*), which closely resemble human *ZBTB16* (Aleksejeva *et al.* 2016). Consistent with observations in primary CECs, cortisol also increased the expression of *zbtb16* in zebrafish after 6 h, with the *zbtb16a* variant increasing ∼three-fold and *zbtb16b* increasing ∼1.5-fold (Supplementary Fig. S6A). To determine the role of Zbtb16, we next created double knockdowns at the F0 generation (crispants) using RNPs targeting *zbtb16a* and *zbtb16b*, in which potential indels were visualized in shifting of melt curves (Supplementary Fig. S6B; Kroll *et al.* 2021). We initially established crispants for the paralogs separately (Supplementary Fig. S6C and D). It was evident that *zbtb16a* was the most responsive paralog. However, there was no significant difference in *fkbp5* mRNA induction between wild-type larvae and single crispants, possibly due to genetic compensation (Supplementary Fig. S6E). As Zbtb16 induction from either paralog may be sufficient to modulate the transcriptional activity of GR, double mutants (*zbtb16ab*) were generated.

**Figure 4:**
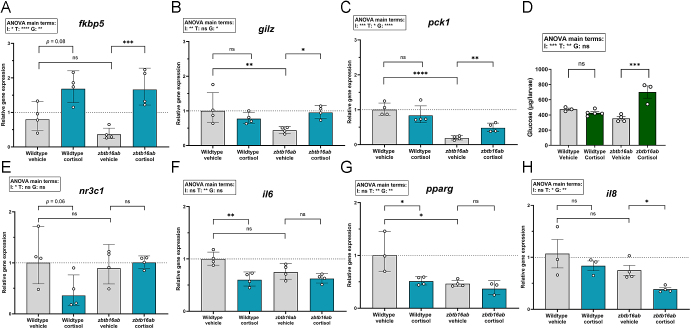
Modulation of GR-mediated transactivation and transrepression of genes by *zbtb16ab* in zebrafish larvae. Five dpf WT zebrafish larvae and *zbtb16ab* crispants (*n* = 10–30) were treated with vehicle or 5 μg/mL cortisol for 20 h. Cortisol-induced GR target genes are indicated in the first panels at the upper row: (A) *fkbp5* (*P* = 0.0002), (B) *gilz* (*P* = 0.01), (C) *pck1* (*P* = 0.001). (D) Total glucose levels (mean ± S.E.M) from whole-body lysates of either 5 dpf WT zebrafish larvae or *zbtb16ab* crispants, with increased levels in the cortisol-treated crispants (*P* = 0.0002). The lower row shows the relative gene expression of GR-repressive genes: (E) *nr3c1*, (F) *il6*, (G) *pparg*, (H) *il8* (*P* = 0.02). The relative gene expression was normalized to *actb* as a housekeeping gene. Data are presented in geometric means ± geometric S.D. G: genotype, I: interaction, ns: not significant, T: treatment.

We then assessed GR-mediated transactivation of target genes in the *zbtb16ab* crispants. At 5 dpf, induction of GR target genes showed interaction effects for cortisol and genotype. In the *zbtb16ab* crispants, ∼five-fold increase in *fkbp5* transcript abundance was observed after cortisol treatment (Fig. 4A). By comparison, there was only a ∼two-fold induction of *fkbp5* mRNA in wild-type larvae (Fig. 4A), which only reached borderline significance (*P* = 0.08), perhaps due to the relatively long treatment (compare with Supplementary Fig. S6E for 6 h incubation). This exaggerated cortisol effect in the absence of *zbtb16* in zebrafish larvae corresponded to what we observed in the *ZBTB16*-silenced primary CECs (Fig. 2A) and *ZBTB16*-silenced HUVECs (Supplementary Fig. S5B), suggesting that this Zbtb16-mediated GR transcriptional restriction is a conserved response. Other GR target genes showed a similar exaggerated transactivation upon *zbtb16* knockdown. Expression of *gilz* showed a ∼two-fold increase with cortisol compared to vehicle, but only in the *zbtb16ab* crispants (Fig. 4B). Similar to *gilz*, *pck1* expression increased ∼three-fold with cortisol treatment, exclusively in the double mutants (Fig. 4C).

Subsequently, we studied the physiological significance of Zbtb16 on GR transactivation by measuring glucose levels. The enzyme phosphoenolpyruvate carboxykinase (*pepck/pck1*) is a GR target gene that is induced upon stress or cortisol treatment to increase hepatic gluconeogenesis (Kuo *et al.* 2015). Indeed, in agreement with the *pck1* expression results, total glucose levels in *zbtb16ab* crispant larvae increased with cortisol treatment (Fig. 4D), whereas we did not see an elevation of total glucose levels in cortisol-treated WT larvae (Chatzopoulou *et al.* 2015, Faught & Vijayan 2022). Taken together, these results suggest that Zbtb16 limits GR-transactivation-dependent processes in zebrafish.

Next, we assessed whether Zbtb16 also modulated GR-mediated transcriptional repression of *nr3c1, il6*, *pparg*, and *il8*. Overall, we found that in crispants for two genes (*nr3c1* and *il8*), the repression upon cortisol was reduced, and for two genes (*il6* and *pparg*), a similar trend was visible, but this did not reach significance. In detail, the expression of *nr3c1* decreased by half in WT larvae treated with cortisol, and this downregulation was abolished in the *zbtb16ab* crispants (Fig. 4E), suggesting Zbtb16 is necessary for GR-mediated homologous downregulation in zebrafish. The repression of *il6* mRNA levels in cortisol-treated WT larvae seemed attenuated in *zbtb16ab* crispants, although the interaction term in the two-way ANOVA did not reach significance (Fig. 4F). The analysis of *pparg* mRNA transcript abundance showed main effects of both cortisol and gene modification, but no significant interaction between the two (Fig. 4G). In contrast, cortisol did not alter expression of *il8* in WT larvae, but in *zbtb16ab* knockdown larvae, the transcript abundance of *il8* was decreased upon cortisol treatment (Fig. 4H).

Overall, these expression data suggest that also in zebrafish larvae, Zbtb16 may attenuate transactivation of specific genes, while it may potentiate transrepression in a gene-specific manner.

### Zbtb16 potentiates glucocorticoid-induced anti-inflammatory action

**Figure 5:**
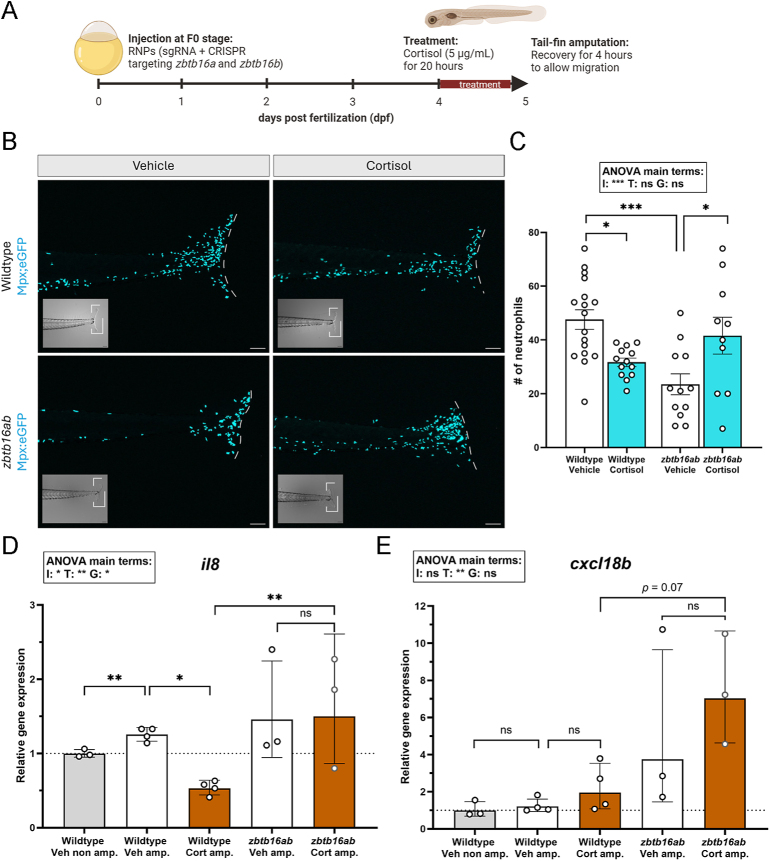
Larvae lacking *zbtb16ab* show abrogated cortisol effects on leukocyte migration. (A) Summary of the experimental design for tail fin amputation. To generate F0 mutants, the RNPs (comprised of sgRNA, crRNA targeting *zbtb16a* and *zbtb16b*, and preassembled Cas9) were injected in the yolk at the single-cell stage. At 4 dpf, the larvae were treated with vehicle (Veh) or 5 μg/mL cortisol (Cort) for 20 h before tail fin amputation was performed. To allow the migration of macrophages and neutrophils to the wound site, the larvae were allowed to recover for 4 h, with the appropriate treatments maintained, after which they were fixed in 4% paraformaldehyde overnight at 4°C to quantify leukocyte migration or at −80°C to assess the GR-induced repressive genes. (B) Representative images of 5 dpf *Tg (mpx:GFP* (wild-type/WT) or *Tg (mpx:GFP zbtb16ab* (double crispants), treated with either vehicle or 5 μg/mL cortisol. The neutrophils were visualized using GFP fluorescence (cyan). Scale bar: 100 μm. The white dashed lines indicate the end of the tail fin. (C) The number of mpx + neutrophils (mean ± S.E.M) in the wounded area at the tail fin (*n* = 10–30) decreased with cortisol (*P* = 0.02) and was further lowered in the crispants without cortisol (*P* = 0.0004). The decreased number of mpx + neutrophils increased in crispants when cortisol was present (*P* = 0.03). Neutrophil-specific chemoattractant gene expression (geometric means ± geometric S.D): (D) *il8* and (E) *cxcl18b*. G: genotype, I: interaction, non-amp: non-amputation, T: treatment, wt: wild-type.

The ability of Zbtb16 to potentiate transcriptional repression of GR target genes suggests that the anti-inflammatory properties of glucocorticoids may also be modulated by Zbtb16. Zebrafish are an ideal model to study the dynamics of the innate immune system due to their optical transparency at larval stages, which allows for visualization of leukocytes using transgenic lines with fluorescently labeled immune cell types (Xie *et al.* 2020). Therefore, to test whether there was immunological significance to Zbtb16-induced potentiation of GR transrepression, we quantified the migration of macrophages and neutrophils to a localized area of inflammation after tail fin amputation (Fig. 5A).

Cortisol reduced the migration of both neutrophils (Fig. 5B) and macrophages (Supplementary Fig. S7A) towards the site of amputation in WT larvae, which is consistent with previous studies and the generally known anti-inflammatory effect of glucocorticoids (Chatzopoulou *et al.* 2016, Xie *et al.* 2019). In the absence of cortisol, the number of neutrophils that was localized at the amputated site was ∼two-fold lower in the *zbtb16ab* crispants compared to WT larvae (Fig. 5C). This indicates that Zbtb16 may act to suppress the innate immune system during inflammation. Interestingly, upon cortisol treatment, the number of neutrophils localized at the wound site increased in the *zbtb16ab* knockdown larvae compared to vehicle controls (Fig. 5C). This suggests that *zbtb16* is necessary for the cortisol-induced anti-inflammatory effects with regard to neutrophil migration. These data indicate that knockdown of *zbtb16* abolishes GR-induced anti-inflammatory effects on neutrophil migration. Meanwhile, macrophage migration was also reduced upon knockdown of *zbtb16ab* compared to vehicle-treated WT larvae, and the number of macrophages did not differ after cortisol treatment (Supplementary Fig. S7B). Given the intrinsic effects of the knockdown, this observation for macrophages in *zbtb16ab* knockdown zebrafish larvae may reflect a ‘bottom-effect’, in that migration may have been fully inhibited already, and no additional cortisol effect was possible.

To better understand the inflammatory responses associated with cortisol-induced *zbtb16* expression, we next examined the transcriptional regulation of several key chemoattractant genes. It has previously been established that Il8 (Cxcl8a) and Cxcl18b (Cxcl-clc) are important factors for stimulating neutrophil migration, while Ccl2 (Mcp-1) and Cxcl-11aa (Cxcl11.1) are important for macrophage migration (Xie *et al.* 2019). In WT larvae that were subjected to tail fin amputation, a modestly increased transcript abundance of *il8* was observed, but this increase was substantially lowered, more than half, when treated with cortisol (Fig. 5D). This is consistent with previous findings (Xie *et al.* 2019), and reduction of these chemokines by cortisol is thought to be a key mechanism of glucocorticoid-induced anti-inflammatory action. However, in *zbtb16ab* crispants, *il8* mRNA levels were remarkably increased under vehicle conditions and were no longer suppressed by cortisol (Fig. 5D). Similarly, an increased expression of *cxcl18b* in *zbtb16ab* crispants was observed independent of the amputation. The high variation observed in the crispants precluded conclusions of the effects of cortisol on *cxcl18b* expression (Fig. 5E). The induction of *ccl2* mRNA by cortisol was less apparent in *zbtb16ab* crispants (Supplementary Fig. S5C).

Taken together, these data point to an intrinsic effect of Zbtb16 on the immune system, and to Zbtb16 as a key mediator of GR-induced anti-inflammatory action, likely by potentiating GR-mediated transrepressive effects.

### Dexamethasone increases ZBTB16 protein expression in FLS from osteoarthritis patients

**Figure 6:**
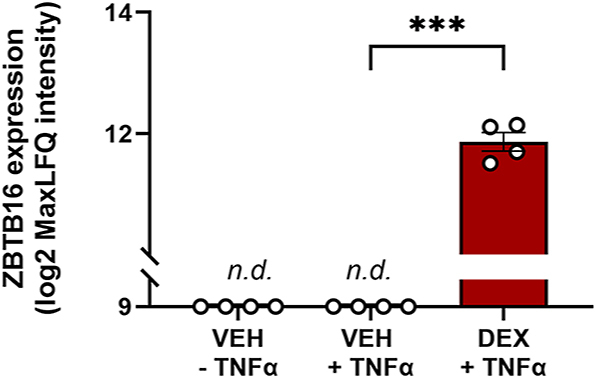
Dexamethasone upregulates ZBTB16 protein expression in FLS derived from osteoarthritis patients. ZBTB16 protein was detected in patient-derived FLS only after treatment with dexamethasone (Dex) (adj. *P* = 0.0002), whereas stimulation by TNF-α shows no effect. Data are presented as mean ± S.E.M. n.d.: not detected, veh: vehicle.

Our observation showed that GR-induced *zbtb16* expression can be involved in anti-inflammatory glucocorticoid effects by enhancing GR-mediated repressive effects. Meanwhile, ZBTB16 restrains glucocorticoid effects that are due to GR’s transactivation activity. This may be relevant from a human clinical perspective, given that the latter have been held responsible for undesired effects of synthetic glucocorticoids that are used in the clinic (De Bosscher *et al.* 2010, De Bosscher *et al.* 2014). To test whether ZBTB16 protein induction by glucocorticoids can occur in a clinically relevant cell population, we used available FLS derived from four osteoarthritis patients. These FLS were incubated with and without the synthetic glucocorticoid dexamethasone, in the presence of a pro-inflammatory stimulus, TNF-α. Using mass spectrometry-based proteomics, we found that dexamethasone significantly upregulated ZBTB16 protein, while levels were undetectable in the vehicle group (Fig. 6). TNF-α stimulation alone did not influence ZBTB16 expression (Fig. 6). This suggests that ZBTB16 may modulate the efficacy and selectivity of synthetic glucocorticoids in the clinical setting.

## Discussion

**Figure 7:**
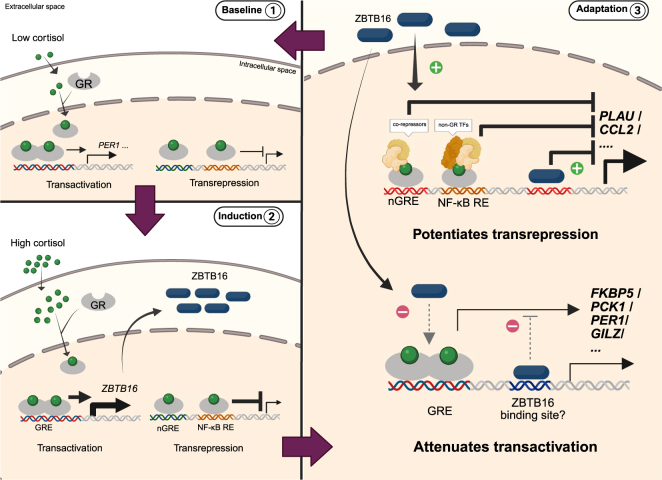
Schematic figure of cortisol-induced ZBTB16 as part of intracellular feedback regulation of GR signaling. Baseline 1: Under low cortisol baseline condition, there is low basal GR transcriptional activity. Induction 2: Under stimulated (high cortisol) conditions, GR induces target genes via transactivation, including *ZBTB16*, and represses other GR target genes. Adaptation 3: Cortisol-induced ZBTB16 protein modulates GR-mediated transcriptional activities by potentiating transrepression and attenuating transactivation effects in a gene-specific manner. This orchestrated activity of glucocorticoid-induced ZBTB16 restores the cellular condition to its baseline state and therefore may be part of an intracellular negative feedback loop. Thick arrows indicate increased transcriptional activity, thin arrows indicate normal or reduced transcriptional activity, and the dashed arrows in gray indicate potential mechanisms. GR: glucocorticoid receptor, GRE: glucocorticoid response elements, nGRE: negative glucocorticoid response elements, NF-κB RE: NF-κB response elements, TFs: transcription factors. Created in BioRender. Galuh, S. (2025) https://BioRender.com/i82d546.

Our findings demonstrate the ability of cortisol-induced ZBTB16 to orchestrate transcriptional activities in a gene-specific manner, with consequences for the functional outcomes of GR stimulation in very diverse contexts. Lowering the expression of *ZBTB16* potentiated GR-dependent effects on endothelial cell barrier function in human CECs and cortisol-induced metabolic alterations in zebrafish larvae. In contrast, we observed attenuated GR-dependent transrepression, suggesting that transactivation and transrepression activities are decoupled in the absence of ZBTB16. Indeed, *zbtb16* knockdown abrogated the GR-dependent effect on leukocyte migration in a zebrafish model, pointing to a key role for Zbtb16 in modulating the immune-suppressive effects of cortisol. Altogether, cortisol-induced ZBTB16 may be part of an intracellular GR negative feedback loop to redirect the cellular response toward baseline conditions, dampening both the direct activation of GR effects (e.g., glucose secretion) and (steroid-independently) activated transcription factor pathways (Fig. 7; Sapolsky *et al.* 2000). These findings may be clinically relevant given the induction of ZBTB16 protein in human FLS by dexamethasone.

ZBTB16 has intrinsic effects in the innate immune system, as shown previously in mice (Mao *et al.* 2016) and supported by our data in zebrafish larvae. ZBTB16 may act as part of transcriptionally active protein complexes (Martin *et al.* 2003) and as a DNA-binding protein (Sadler *et al.* 2015). Its anti-inflammatory effects involve transrepressive effects on gene expression and interactions with co-repressors HDAC3 and with NF-ĸB (Sadler *et al.* 2015). ZBTB16 also controls developmental processes in different cell types, including NKT cells (Mao *et al.* 2016). Yet, in contrast to NKT cells, in many tissues, *ZBTB16* expression is very low under basal conditions. GR activation can result in a potent increase in ZBTB16 expression, as evidenced both in primary CECs and clinical samples from osteoarthritis patients. It can therefore be considered as one of the effector mechanisms of anti-inflammatory glucocorticoids, taking effect some hours after initial GR activation. The roles of ZBTB16 in developmental processes and as a mediator of glucocorticoid effects converged in a study that identified ZBTB16 as a mediator of glucocorticoid effects on neuronal differentiation (Krontira *et al.* 2024).

Of note, the induction of ZBTB16 for the anti-inflammatory effects of GR depends on transactivation. Several studies identified multiple GR-binding peaks in human cells, at about 10 kb upstream of the transcription start site (Vockley *et al.* 2016) or within intronic regions of the *ZBTB16* gene after being treated with dexamethasone (Karagiannopoulos *et al.* 2023, Prekovic *et al.* 2023). In mice, a GRE half site in the promoter of the gene may be necessary for GR-dependent gene induction (Chen *et al.* 2014). This transactivation is reminiscent of other mediators of glucocorticoid anti-inflammatory effects, like *GILZ* (D'Adamio *et al.* 1997) and *DUSP1* (Abraham *et al.* 2006). Together, these data reemphasize the notion that both transactivating and transrepressive effects of GR are important for its anti-inflammatory action (Sundahl *et al.* 2015).

The role of ZBTB16 as a mediator of GR effects in the immune response is in stark contrast with its role in other processes, where it limits the effects of GR activation. A previous study showed that glucocorticoid-induced ZBTB16 limited GR-mediated apoptosis (Wasim *et al.* 2010). Another study in human insulin-producing beta cells showed that ZBTB16 overexpression attenuated the effect of glucocorticoids on suppression of mitochondrial activity (Karagiannopoulos *et al.* 2023). In our data, we observed that ZBTB16 limited the functional effect of GR in human endothelial cells, as well as total glucose release in zebrafish larvae, even if ZBTB16 can have intrinsically stimulating effects on gluconeogenesis in the mouse (Chen *et al.* 2014). A similar ‘restraining’ effect was observed for mineralocorticoid receptor (MR)-mediated action on sodium transport in kidney epithelial cells, where ZBTB16 mRNA was directly induced by MR over the course of several hours (Náray-Fejes-Tóth *et al.* 2008). Thus, for several effects of glucocorticoids, likely transactivation-dependent, ZBTB16 acts to restrain rather than mediate the GR-dependent effects.

Given that both GR and ZBTB16 are transcriptionally active proteins, it seems likely that the modifying effect of ZBTB16 on GR functionality involves transcription. Although the measurement of nascent RNA would be needed to establish this with more certainty, our data indicated that knockdown of ZBTB16 potentiated GR-mediated transactivation and reduced GR-mediated transrepression of some GR-dependent target genes. Our transcription data from zebrafish larvae showed an attenuation of homologous downregulation of *nr3c1* in the crispants. One may envisage that this leads to stabilization of GR protein and that this may contribute to the enhancement of GR-mediated transactivation. However, the gene-specific effects of *zbtb16ab* knockdown and the attenuated transrepression suggest that this is not the dominant mechanism underlying our data. Experimental evidence for GR stability is not easily achieved, as specific antibodies against GR are lacking in zebrafish. Our mixed data are in line with an earlier study looking at partial knockdown of *ZBTB16* in human cells (Karagiannopoulos *et al.* 2023). Our functional knockout data from zebrafish larvae confirmed that for several genes, *zbtb16* restrained GR-dependent transactivation and that for some genes, it was necessary for GR-dependent transrepression. Yet, the extent of this ‘dissociative’ effect will require whole-genome analysis of GR transcriptional targets. Conceivably, ZBTB16 acts as a corepressor recruited in transcriptionally active GR complexes. Our PLA data, however, provided no strong evidence that both proteins are not in close vicinity. This, in turn, is in agreement with a recent study showing that, using rapid immunoprecipitation mass spectrometry (RIME), ZBTB16 did not co-immunoprecipitate with the GR (Prekovic *et al.* 2023). These data indicate that ZBTB16 may bind to independent binding sites in select GR target genes to modify GR-mediated transcription effects (Mao *et al.* 2016). ChIP-seq data for both factors in the same cell type may confirm this notion in later studies.

Overall, our data are compatible with ZBTB16 being an intracellular modulator of GR function, perhaps limiting activating effects of cortisol (e.g., glucose production) and facilitating repression of transcriptional programs that were activated via other signaling pathways. Such a scenario would facilitate a return of the cell to baseline conditions after an initial transcriptional response to (immune) challenges that evoke an HPA axis response: protein induction driven both by cortisol and by transactivation partners of GR are both curtailed by ZBTB16 in the time frame of hours after initial GR activation. Such a ‘second wave’ modulation of GR activity by its own transcriptional target is reminiscent of the negative feedback that GR-induced *FKBP5* exerts on overall GR signaling (Zannas & Binder 2014). Of note, this type of modulation also provides a cross-talk mechanism between GR and MR, which also mediate cortisol effects in many cell types. MR can regulate the expression of FKBP51 and ZBTB16, and its effects were found to be limited by ZBTB16 (Náray-Fejes-Tóth *et al.* 2008, van Weert *et al.* 2019, Hartmann *et al.* 2021). Adding to the complexity of autoregulatory mechanisms of GR signaling, the induction of the negative GR regulator FKBP51 was also restrained by ZBTB16, indicating several (interactive) feedback loops may interact to fine-tune the action of GR.

ZBTB16 may be relevant in the setting of glucocorticoid therapy, given that its presence in the cells derived from osteoarthritis patients was dependent solely on dexamethasone treatment. In the clinic, glucocorticoids can be lifesaving anti-inflammatory drugs, but they are also risk factors for a broad range of diseases. In fact, our initial experiments were based on the hypothesis that ZBTB16 may be a mediator of glucocorticoid effects in central serous chorioretinopathy (CSC). CSC is an eye disease strongly associated with glucocorticoid exposure and has therefore been considered a consequence of the pleiotropic role of glucocorticoids (Brinks *et al.* 2021). Separating necessary therapeutic results from negative side effects has been a longstanding goal in glucocorticoid research (De Bosscher *et al.* 2010, Meijer *et al.* 2023). The fine-tuning of GR activity by ZBTB16 in fact works in the direction of ‘dissociated ligands’ of GR: drugs that retain anti-inflammatory efficacy while reducing side effects, such as insulin resistance (Schäcke *et al.* 2004). It is highly uncertain whether targeting ZBTB16 is a viable option to increase the therapeutic effect of glucocorticoids because it also has many intrinsic effects (Seda *et al.* 2017). Nevertheless, understanding how ZBTB16 fine-tunes the transcriptional GR response may point to new avenues in drug development aimed at reducing the sometimes debilitating side effects of glucocorticoids while retaining clinical efficacy.

## Supplementary materials



## Declaration of interest

OCM receives funding from Corcept Therapeutics, which develops GR modulators for clinical use. SG, EF, IK, LLK, JB, EvD, DE, RO, MS, and CJFB have nothing to disclose.

## Funding

SG was supported by a kind donation to the Bontius Foundation LUMC via RO. EF was supported by an NWO Veni grant and a fellowship from the Canadian Institutes of Health Research (CIHR). LLK was supported by Fonds Wetenschappelijk Onderzoekhttps://doi.org/10.13039/501100003130 (1257523N). IK was supported by the following foundations: Landelijke Stichting voor Blinden en Slechtzienden, Algemene Nederlandse Vereniging ter Voorkoming van Blindheid, Stichting Blinden-Penninghttps://doi.org/10.13039/501100010373, and Oogfonds, which contributed through UitZicht (grants UZ2021-27 and UZ2022-25), Rotterdamse Stichting Blindenbelangenhttps://doi.org/10.13039/501100010372 (B20220071), the Dutch Diabetes Research Foundationhttps://doi.org/10.13039/501100003092 (grant 2022.22.009), and through funding of the Amsterdam UMC Foundation. These funding organizations had no role in the design or conduct of this research. They provided unrestricted grants. EVD and CJFB received support from the following foundations: Stichting Macula Fonds (CJFB), Retina Nederland Onderzoek Fonds (CJFB), Stichting Blinden-Penning (EVD, CJFB), Oogfonds (EVD), Algemene Nederlandse Vereniging ter Voorkoming van Blindheid (EVD, CJFB), and Landelijke Stichting voor Blinden en Slechtzienden, which contributed through UitZicht, as well as Rotterdamse Stichting Blindenbelangen (EVD, CJFB), Stichting Leids Oogheelkundig Ondersteuningsfonds (EVD, CJFB), Haagse Stichting Blindenhulp (CJFB), Stichting Ooglijders (EVD, CJFB), Stichting Steunfonds UitZicht (EVD), Stichting Blindenhulp (EVD), ZonMw VENI Grant (CJFB), and Gisela Thier Fellowship of Leiden University (CJFB). These funding organizations had no role in the design or conduct of this research. They provided unrestricted grants. DE was supported by grants from the Fund for Scientific Research Flanders, the Research Council of Ghent University, and Stichting tegen Kanker.

## Data availability

The datasets generated in this current study are available upon reasonable request from the corresponding author.
