# Supplementary material for: The glucocorticoid receptor is affected by its target ZBTB16 in a dissociated manner
Source: J Endocrinol. 2025 Jul 4;266(1):e240283. doi: 10.1530/JOE-24-0283 (PMC12231182; doi:10.1530/JOE-24-0283)
Supplement: Supplementary file 1 [file supplementary_materials.pdf]

Supplementary Information

SI 1. Ethics statement

The study protocol for post-mortem eye donor was approved by the local ethics committee of LUMC and followed the Declaration of Helsinki on the use of human material for research. The donors gave written informed consent for organ and tissue donation, in accordance with Dutch laws. The study protocol involving osteoarthritis patients was approved by the Ghent University Hospital Ethics Committee (BC-06496). Informed consent was obtained from all participating patients.

The human serum used in the siRNA-mediated knockdown of *ZBTB16* in primary CECs was approved by the Medical Ethical Review Committee of the Academic Medical Center Amsterdam. Subjects gave informed consent for the use of serum and samples were stored anonymously. Human serum was collected according to the principles of conduct for research integrity in the Amsterdam UMC Research Code (https://www.amsterdamumc.org/en/research/research-code.htm).

SI 2. Preparation of LC-MS/MS samples

The lysate was transferred to a 96-well PIXUL plate and sonicated with a PIXUL Multisample sonicator (Active Motif) for 5 minutes with default settings (Pulse 50 cycles, PRF 1 kHz, Burst Rate 20 Hz). Next, samples were centrifuged for 15 minutes at 2,204 xg at RT to remove insoluble components. Proteins were reduced and alkylated by addition of 10 mM Tris(2-carboxyethyl)phosphine hydrochloride and 40 mM chloroacetamide and incubation for 10 minutes at 95°C in the dark. Phosphoric acid was added to a final concentration of 2.75% and subsequently samples were

diluted 7-fold with binding buffer containing 90% methanol in 100 mM TEAB, pH 7.55 (binding buffer). After loading the samples to S-trap micro columns (Protifi) using centrifugation for 1 min at 1,500 xg, the columns were washed 3 times with 150 µl binding buffer using the same centrifugation settings. Digestion of the proteins was done by addition of 20 µl 50mM TEAB containing 1 µg of trypsin and incubation overnight at 37°C. Peptides were eluted in 3 times, first with 40 µl 50 mM TEAB, then with 40 µl 0.2% formic acid (FA) in water and finally with 40 µl 0.2% FA in water/acetonitrile (ACN) (50/50, v/v). Eluted peptides were dried completely by vacuum centrifugation. The dried peptides were redissolved in 20 µl 0.1% TFA in water/ACN (98/2, v/v), diluted 100-fold with 0.1% FA of which 20 µl was loaded on Evotips (Evosep, P/N EV2011, Odense, Denmark) according to the manufacturer's instructions. All loaded Evotips were stored in 0.1% FA at 4°C until LC-MS/MS analysis could be started.

SI 3. LC-MS/MS analysis

Samples were run in data-independent parallel accumulation serial fragmentation (DIA-PASEF) mode on an Evosep One LC-system (Evosep) in-line connected to a timsTOF SCP (Bruker, Belgium). Peptides were analyzed with the 20 SPD whisper method using the Aurora Gen3 Elite column (15cm x 75 µm I.D., 1.7µm beads, Evosep), heated to 50°C. Peptides were eluted from the column through the predefined 20SPD whisper gradient consisting of 0.1% FA in LC-MS-grade water as solvent A and 0.1% FA in ACN as solvent B. Eluting peptides were measured in positive polarity with a full-scan range of 100 m/z to 1700 m/z. The trapped ion mobility spectrometry (TIMS) was operated at a fixed duty cycle close to 100%, a ramp and accumulation time of 100 ms, ranging from $1/K_0$ =0.64 $Vscm^2$ to $1/K_0$ =1.50 $Vscm^2$.

Collision energy was linearly ramped as a function of the inverse mobility from 20 eV at $1/K_0$=0.60Vscm^2 to 59 eV at $1/K_0$ = 1.60 Vscm^2. A DIA-PASEF mass range of 400 Da to 1000 Da was used in a mobility range of $1/K_0$ =0.64 Vscm^2 to $1/K_0$ =1.37 Vscm^2 using a window size of 25 Da according to Table S5, resulting in a cycle time of 0.96s.

SI 4. LC-MS/MS data analysis

LC-MS/MS runs of all samples were searched together using the DiaNN algorithm (version 1.8.1) (Demichev *et al.*, 2020). Spectra were searched against the human protein sequences in the Swiss-Prot database (database release version of 2023_08), containing 20,423 sequences. Enzyme specificity was set as C-terminal to arginine and lysine, also allowing cleavage at proline bonds with a maximum of 2 missed cleavages. Variable modifications were set to oxidation of methionine residues and acetylation of protein N-termini. Fixed modification was set to carbamidomethylation of cysteine residues. Default settings were mainly used, except for the addition of a 400-1000 m/z precursor mass range filter and MS1 and MS2 mass tolerance was set to 15 and 20 ppm, respectively. Further data analysis of the shotgun results was performed with an in-house script in the R programming language (version 4.2.2). Protein expression matrices were prepared as follows: the DIA-NN main report output table was filtered at a precursor and protein library q-value cut-off of 1% and only proteins identified by at least one proteotypic peptide were retained. After pivoting into a wide format, iBAQ intensity columns were then added to the matrix using the DIAgui's R package get_IBAQ function (Gerault *et al.*, 2024). Groups of 7,650 proteins were reliably quantified using protein group maximum likelihood quantification (PG.MaxLFQ), with at least 3 valid values in one of the experimental conditions.

Table S1. crRNA specific sites targeting *zbtb16a* and *zbtb16b*

Ensembl	Target #	crRNA sequence	Exon
ENSDART00000184419.1 (*zbtb16a*)	Target 1	CACGCTCCAAGCTAAAGTAG(AGG – PAM site)	2
	Target 2	TCCTAGACAGTCTGCGCCTA(AGG – PAM site)	3
	Target 3	AGAACTCGCACTCGAACGGA(TGG – PAM site)	6
ENSDART00000155866.2 (*zbtb16b*)	Target 1	CGCCACGCTCCAAGCCAAGC(TGG – PAM site)	2
	Target 2	TCCGCAGAACTCACAGCCAA(AGG – PAM site)	3
	Target 3	CGTGACGAGAGCACATTGAA(GGG– PAM site)	6

Table S2. Gene-specific primers for human (*Homo sapiens*)

Gene	Forward primer (5' → 3')	Reverse primer (5' → 3')	Ref.
ZBTB16/ PLZF	GTTCCTGGATAGTTTGCG	CATGTCAGTGCCAGTATG	(Brinks *et al.*, 2022)
FKBP5	TGGAAAGAAGTTTGATTCCAGTCAT	CATGGTAGCCACCCCAATGT	(Brinks *et al.*, 2022)
PER1	CACTCCTGCGACCAGGTA	TAGGGGGCCACTCATGTCT	(Brinks *et al.*, 2018)
ITGA10	Proprietary, Gene GlobeID: QT00031150, Qiagen		
ANGPTL4	AGGCAGAGTGGACTATTTG	CCTCCATCTGAGGTCATC	N/A
TSC22D3 (GILZ)	CCAGCGTGGTGGCCATAGA	CACGCTCTAGCTGGGAGTTC	(Brinks *et al.*, 2018)
NR3C1	TGCCTGGTGTGCTCTGATGA	CACATAGGTAATTGTGCTGTCCTT	(Koning *et al.*, 2022)
PLAU	GAGCGACTCCAAACGAACTGT	GTCAGTGCTGGCCTTTCCTC	N/A
CCL2	TCTGTGCCTGCTGCTCATAG	GGGCATTGATTGCATCTGGC	N/A
RND1	CGCAACCCTCCCTTCTGAAT	TGGGCACATAGGTCTCTGGA	N/A
LRP10	CAGACTGTCACCATCAGGTTC	GAGAGGGGAGCGTAGGGTTA	(Brinks *et al.*, 2022)
VWF	GTGCCTGCAACTGTGTCAAC	CAGGGTAGATGGTGCTTCGG	(Brinks *et al.*, 2018)

CD34	GCGCTTTGCTTGCTGAGTTTGC	GCCTCATTGCCATGTTGAGACAC	(Brinks *et al.*, 2018)

Table S3. Gene-specific primers for zebrafish (*Danio rerio*)

Gene	Forward primer (5’ → 3’)	Reverse primer (5’ → 3’)	Ref.
actb	CGAGCAGGAGATGGGAACC	CAACGGAAACGCTCATTGC	(Schaaf *et al.*, 2008)
zbtb16a	CTTGGAGGAGCAATGCCTGA	TTCTGTGCCACCATCGTTCA	N/A
zbtb16b	ACCCAGAAAGAGCGGTGTGAGG	CAGCATGTGCATTCTCAGCCGT	(Aleksejeva *et al.*, 2016)
fkbp5	TCTGCCAGCACAAGATTCGTGAGC	GACCCTGCTTATTCTGATCGGAAA	(Chatzopoulou *et al.*, 2017)
nr3c1	ACAGCTTCTTCCAGCCTCAG	CCGGTGTTCTCCTGTTTGAT	(Alsop and Vijayan, 2008)
pparg	TGCCGCATACACAAGAAGAG	ATGTGGTTCACGTCACTGGA	(Faught and Vijayan, 2019)
pck1	CAGGGCGATCTGGCGTCTCT	CTGCTGTCGATGAACTCCCG	(Chatzopoulou *et al.*, 2017)
il8	TGTGTTATTGTTTTCCTGGCATTTC	GCGACAGCGTGGATCTACAG	(Xie *et al.*, 2019)
cxcl18b	TCTTCTGCTGCTGCTTGCGGT	GGTGTCCCTGCGAGCACGAT	(Xie *et al.*, 2019)
il6	CGCTAAGGCAACTGGAAGAC	CCAGACCACTGGGAAACACT	(Xie *et al.*, 2019)
il1b	TGTGTGTTTGGGAATCTCCA	CTGATAAACCAACCGGGACA	(Xie *et al.*, 2019)

Table S4. Antibodies list for western blot

Target	Type of antibodies	Company	Catalog no. #	Host Species	Working dilution WB	Blocking agent
ZBTB16	Primary	Thermo Fisher Scientific	MA5-15667	Mouse	1:1000	5% w/v BSA in 1X TBS with Tween-20
GR	Primary	Cell Signaling Technology	D6H2L	Rabbit	1:1000	5% w/v BSA in 1X TBS with Tween-20
Beta-Actin	Primary	Abcam	ab8229	Goat	1:1000	5% w/v BSA in 1X TBS with Tween-20
Lamin A/ Lamin C	Primary	Cell Signaling Technology	2032	Rabbit	1:1000	5% w/v BSA in 1X TBS with Tween-20
anti-mouse IgG (H+L) HRP conjugate	Secondary	Promega	W4021	Goat	1:2000	-
anti-rabbit IgG (H+L) HRP Conjugate	Secondary	Promega	W401B	Goat	1:5000	-
anti-goat IgG-HRP	Secondary	Santa Cruz Biotechnology	sc-2020	Donkey	1:5000	-

Abbreviations: BSA, bovine serum albumin; GR, glucocorticoid receptor; HRP, horseradish peroxidase; TBS, tris buffered saline; WB, western blot; ZBTB16, zinc finger and BTB domain containing 16.

Table S5. Dia-PASEF windows

Cycle Id	Start IM [1/K0]	End IM [1/K0]	Start Mass [m/z]	End Mass [m/z]
1	0.64	0.83	400	425
2	0.64	0.85	425	450
3	0.64	0.87	450	475
4	0.64	0.9	475	500
5	0.64	0.92	500	525
6	0.64	0.94	525	550
7	0.64	0.97	550	575
8	0.64	0.99	575	600
1	0.83	1.01	600	625
2	0.85	1.04	625	650
3	0.87	1.06	650	675
4	0.9	1.09	675	700
5	0.92	1.11	700	725
6	0.94	1.13	725	750
7	0.97	1.16	750	775
8	0.99	1.18	775	800
1	1.01	1.37	800	825
2	1.04	1.37	825	850
3	1.06	1.37	850	875
4	1.09	1.37	875	900
5	1.11	1.37	900	925
6	1.13	1.37	925	950
7	1.16	1.37	950	975

8	1.18	1.37	975	1000

Abbreviations: IM: ion mobility, 1/K0: ion mobility at standard pressure and standard temperature, m/z: mass-to-charge

Legends for Supplemental Figures

Fig. S1. The purity of isolated human primary CECs

The purity of isolated primary CECs were confirmed by flow cytometry detecting two endothelial cell markers, CD31 and VE-Cadherin. Figures A-E show flow cytometry density plots of representative donor (donor 1) in different experimental groups. **A:** Unstained CECs (negative control) showed clusters of cells with low CD31 (APC-A) and VE-cadherin (PE-A) expression. **B:** CD31-stained CECs (positive control) showed high expression of CD31. **C:** VE-Cadherin-stained CECs (positive control) showed high expression of VE-Cadherin. **D:** Primary CECs derived from the left eye (LE) had 95,3% positive of CD31 and VE-cadherin. **E:** Primary CECs derived from the right eye (RE) had 88,3% positive of CD31 and VE-cadherin. The endothelial (F-G) gene markers were confirmed across donors, depicting ΔCT values for **F:** *VWF*, and **G:** *CD34*.

Fig. S2. Relative gene expression of GR-mediated transcriptional activities.

Primary CECs derived from 5 post-mortem eye donors were transfected with SMARTpool mix si*ZBTB16*, siNT, or not transfected with siRNA at all (non-treated group) for 48 hours. Cortisol-induced glucocorticoid receptor target genes were measured after treatment with 100 nM cortisol (Cort) for 4 hours. First row shows **A:** *FKBP5*, **B:** *PER1*, **C:** *ITGA10*, **D:** *ANGPTL4*. Second row shows **E:** *TSC22D3 (GILZ)*, **F:** *CCL2*, **G:** *PLAU*, **H:** *RND1*, and **I:** *NR3C1* at the last row. The gene expression was relative to vehicle from non-treated group per donor. The Ct value of target gene was normalized to *LRP10* as a housekeeping gene. The dots in the bar graph represent all technical replications from all donors. ns: not significant.

Fig. S3. Silencing efficiency of *ZBTB16* in primary CECs from each post-mortem eye donor. A: The relative expression of *ZBTB16* mRNA level at 48 hours post-siRNA transfection in primary CECs from each donor (geometric means ± geometric S.D): donor 1 (p = 0.001), donor 3 (p = 0.04), donor 4 (ns), donor 5 (p = 0.005), donor 6 (p = 0.05). Statistical differences were determined using an unpaired t-test. **B:** No correlation was observed between the ratio of the fold change si*ZBTB16*/siNT and the attenuation of the expression of GR target genes.

Fig. S4. ZBTB16 gene and protein expression in HUVECs. **A**: The dose response curve of *ZBTB16* in HUVECs after treatment with vehicle, 1, 10, 100, or 1000 nM cortisol for 6 hours. The EC_{50} concentration was at 22,1 nM. **B**: The ZBTB16 protein expression in HUVECs after treatment with vehicle (veh), 100 nM, or 1000 nM cortisol (cort) for 4, 6, or 24 hours. **C**: Representative images of ZBTB16 immunofluorescence staining after treatment with vehicle or cortisol 100 nM for 24 hours.

Fig. S5. GR-mediated genes expression in *ZBTB16*-silenced HUVECs. HUVECs were treated with either SMARTpool mix siNT or si*ZBTB16* for 48 hours. **A**: The relative expression of *ZBTB16* mRNA level at post-siRNA transfection in HUVECs. Cortisol-induced GR transactivation genes were measured after treatment with 100 nM cortisol (Cort) for 4 hours (upper row): **B:** *FKBP5*, **C:** *PER1*, **D:** *ANGPTL4*, **E:** ITGA10. The lower row shows repressed genes: **F:** *CCL2*, **G:** *NR3C1*, **H:** *RND1*, **I:** *PLAU*. The Ct value of target gene was normalized to *LRP10* as a housekeeping gene. All graphs are presented in geometric means ± geometric S.D. *$p < 0.03$, **$p < 0.002$, ***$p < 0.0002$, ****$p < 0.0001$. FC = fold change, ns: not significant.

Fig. S6. Gene expression levels in single crispants. A: The relative gene expression levels of *zbtb16a* (left) and *zbtb16b* (right) in WT 5 dpf with and without cortisol treatment. **B:** Validation of the *zbtb16ab* crispants RNPs using crRNA target 2. White bars show the average melting temperature of amplicons generated from WT larvae amplicons. The pink bars denote larvae injected with zbtb16a RNPs. The purple bars denote larvae injected with zbtb16b RNPs. Each data point represents a single larva. **C-D:** *zbtb16a/b* gene expression in single crispants (*zbtb16a/b*). **E:** *fkbp5* in single crispants (*zbtb16a/b*). The Ct value of target gene was normalized to *actb*. All graphs are presented in geometric means ± geometric S.D. G: genotype, I: interaction, ns: not significant, T: treatment.

Fig. S7. The macrophage migration in tail-amputated *zbtb16ab* crispants. A: Representative images of 5 dpf *Tg(mpeg1:mcherry-F* (WT)) or *Tg(mpeg1:mcherry-F zbtb16ab* (double crispants)), treated with either vehicle or 5 ng/mL cortisol. The macrophages were visualized by mCherry fluorescence (magenta). Scale bar: 100 µm. The white dashed lines indicate the end of the tail fin. **B:** The number of mpeg+ macrophages (mean ± S.E.M) in the wounded area at the tail fin of WT larvae (n = 10-30) decreased with cortisol (p = 0.02) and lowered more in crispants treated with vehicle (p = 0.1). The low number of mpeg+ macrophages did not differ significantly in crispants when cortisol was present. **C:** The macrophage-specific chemoattractant relative gene expression of *ccl2* did not differ across the groups (geometric mean ± geometric S.D). amp: amputation, cort: cortisol, G: genotype, I: interaction, non-amp: non-amputation, ns: not significant, T: treatment.

Fig. S1.

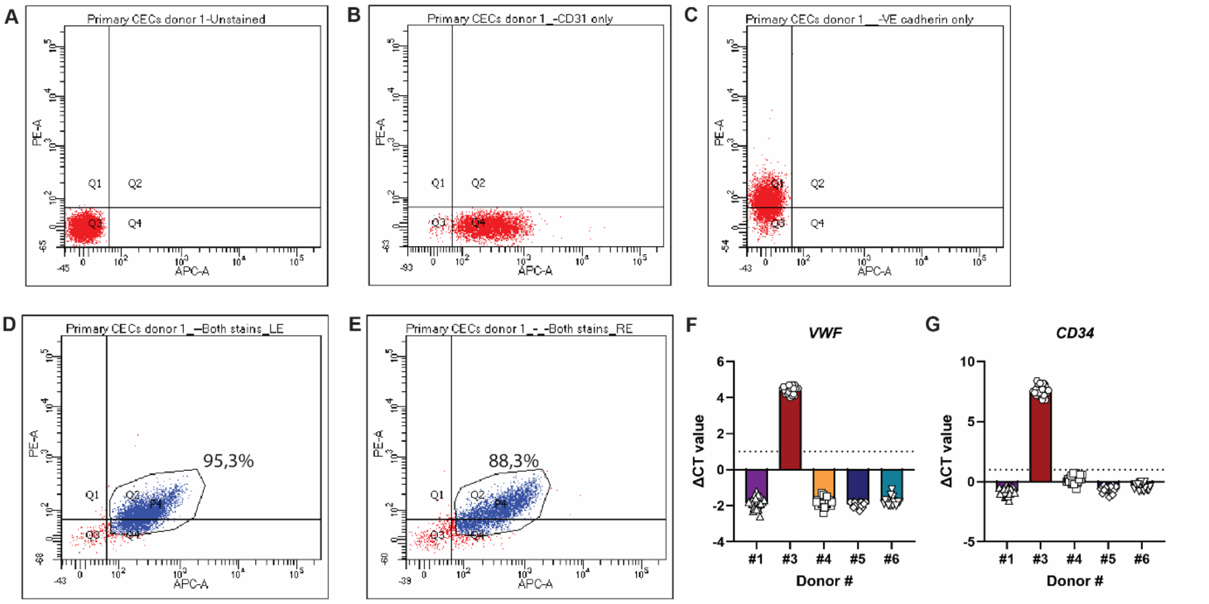
A
Primary CECs donor 1-Unstained
B
Primary CECs donor 1_-CD31 only
C
Primary CECs donor 1__-VE cadherin only
D
Primary CECs donor 1_--Both stains_LE
95,3%
E
Primary CECs donor 1_-_-Both stains_RE
88,3%
PE-A
APC-A
Q1
Q2
Q3
Q4
F
VWF
G
CD34
ΔCT value
#1
#3
#4
#5
#6
Donor #

Fig. S2.

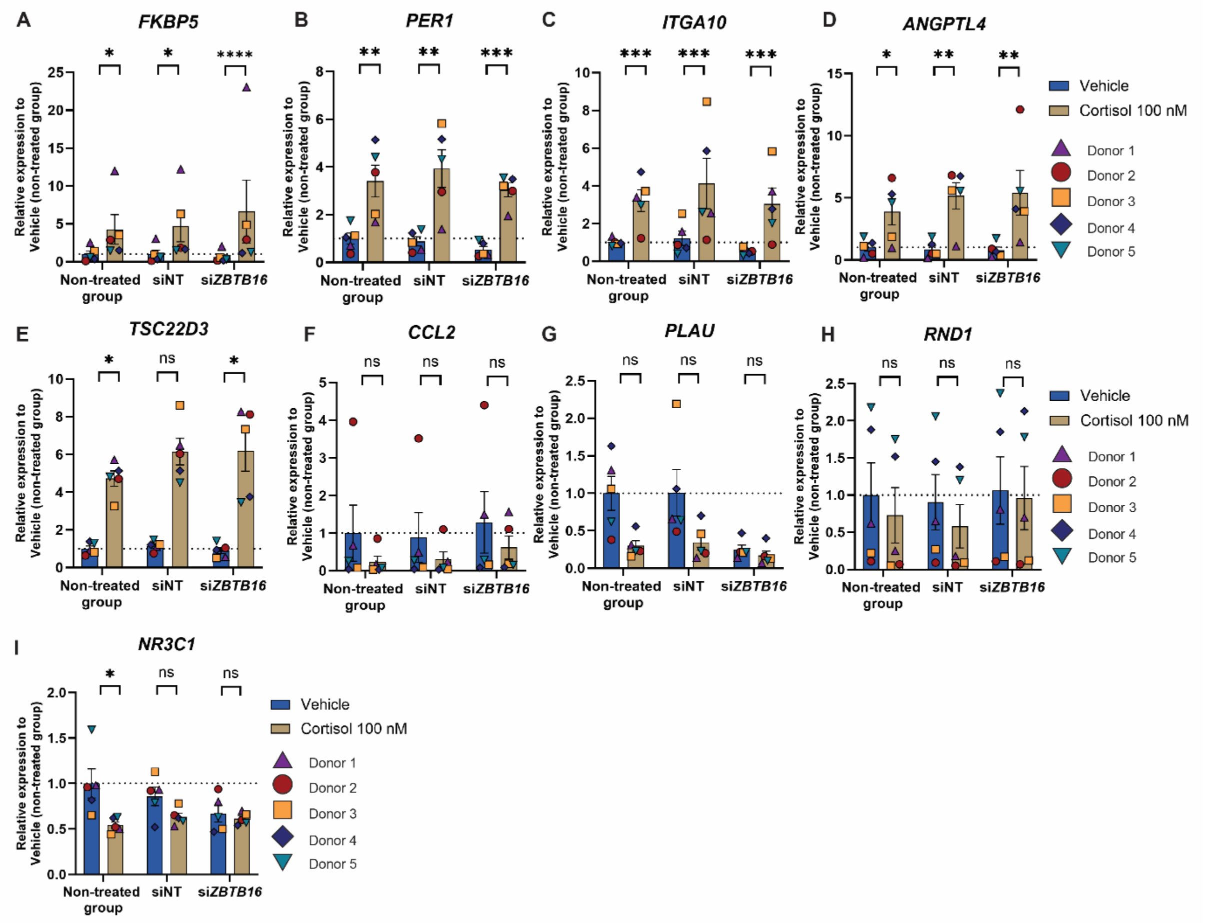
A FKBP5
B PER1
C ITGA10
D ANGPTL4
E TSC22D3
F CCL2
G PLAU
H RND1
I NR3C1
Relative expression to Vehicle (non-treated group)
Non-treated group
siNT
siZBTB16
Vehicle
Cortisol 100 nM
Donor 1
Donor 2
Donor 3
Donor 4
Donor 5
ns

Fig. S3.

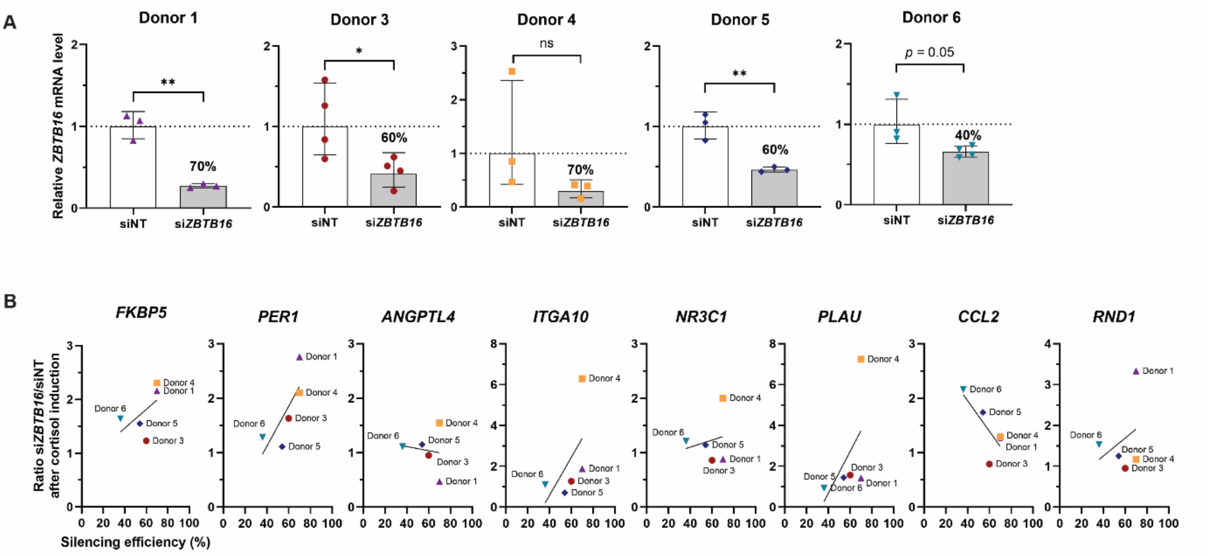

	Silencing efficiency versus ...				
	FKBP5	*PER1*	*ANGPTL4*	*NR3C1*	*CCL2*
R squared (Pearson)	0.30	0.59	0.02	0.04	0.57
P (two-tailed)	0.34	0.13	0.83	0.75	0.14

	Silencing efficiency versus ...		
	ITGA10	*PLAU*	*RND1*
r (Spearman)	0.87	0.56	-0.05
P (two-tailed)	0.10	0.40	>0.99

Fig. S4.

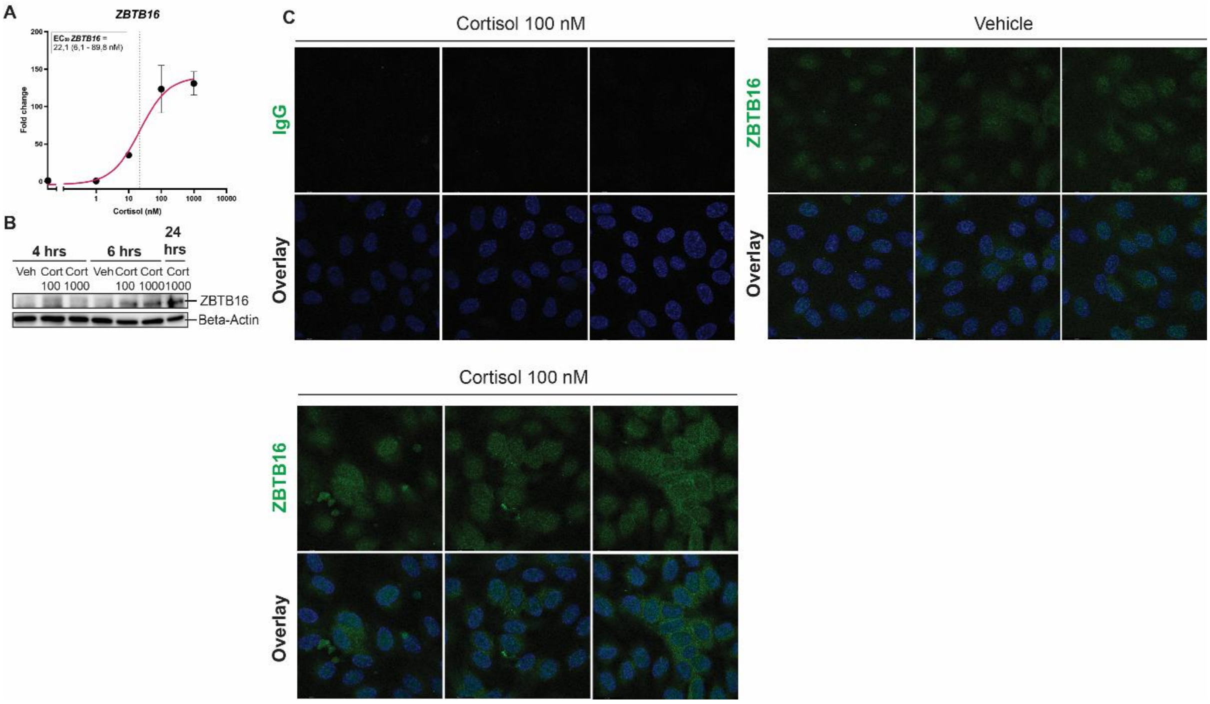
A
ZBTB16
EC50 ZBTB16 =
22,1 (6,1 - 89,8 nM)
Fold change
0
50
100
150
200
1
10
100
1000
10000
Cortisol (nM)
B
4 hrs
6 hrs
24 hrs
Veh
Cort 100
Cort 1000
Veh
Cort 100
Cort 1000
Cort 1000
ZBTB16
Beta-Actin
C
Cortisol 100 nM
IgG
Overlay
Vehicle
ZBTB16
Overlay
Cortisol 100 nM
ZBTB16
Overlay

Fig. S5.

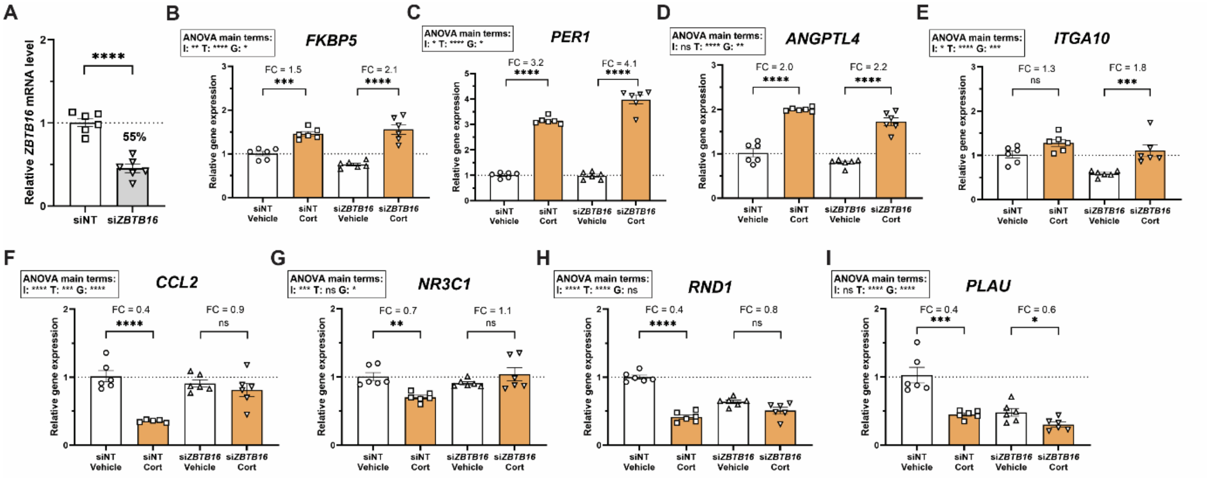
A
Relative ZBTB16 mRNA level
****
55%
siNT
siZBTB16
B
ANOVA main terms:
I: ** T: **** G: *
FKBP5
FC = 1.5
FC = 2.1
C
ANOVA main terms:
I: * T: **** G: *
PER1
FC = 3.2
FC = 4.1
D
ANOVA main terms:
I: ns T: **** G: **
ANGPTL4
FC = 2.0
FC = 2.2
E
ANOVA main terms:
I: * T: **** G: ***
ITGA10
FC = 1.3
FC = 1.8
F
ANOVA main terms:
I: **** T: *** G: ****
CCL2
FC = 0.4
FC = 0.9
G
ANOVA main terms:
I: *** T: ns G: *
NR3C1
FC = 0.7
FC = 1.1
H
ANOVA main terms:
I: **** T: **** G: ns
RND1
FC = 0.4
FC = 0.8
I
ANOVA main terms:
I: ns T: **** G: ****
PLAU
FC = 0.4
FC = 0.6
Relative gene expression
siNT Vehicle
siNT Cort
siZBTB16 Vehicle
siZBTB16 Cort

Fig. S6.

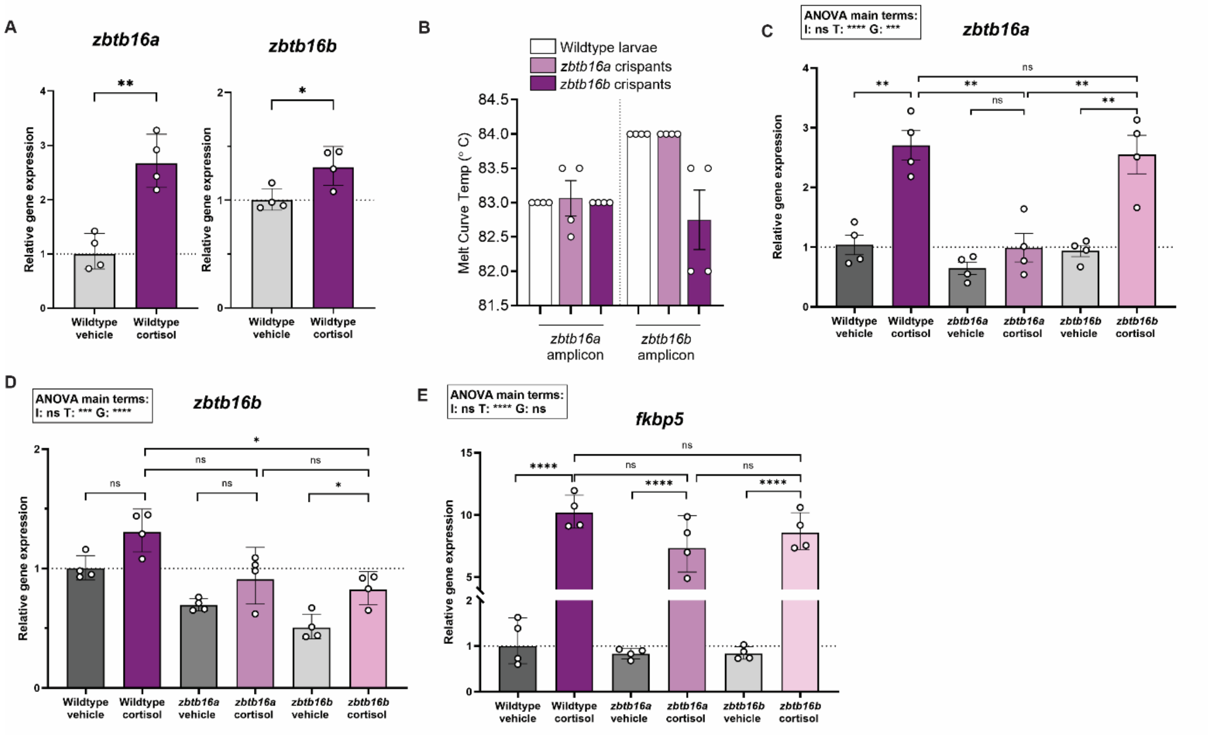
A
zbtb16a
zbtb16b
Relative gene expression
Wildtype vehicle
Wildtype cortisol
B
Wildtype larvae
zbtb16a crispants
zbtb16b crispants
Melt Curve Temp (° C)
zbtb16a amplicon
zbtb16b amplicon
C
ANOVA main terms:
I: ns T: **** G: ***
zbtb16a
zbtb16a vehicle
zbtb16a cortisol
zbtb16b vehicle
zbtb16b cortisol
D
ANOVA main terms:
I: ns T: *** G: ****
zbtb16b
E
ANOVA main terms:
I: ns T: **** G: ns
fkbp5

Fig. S7.

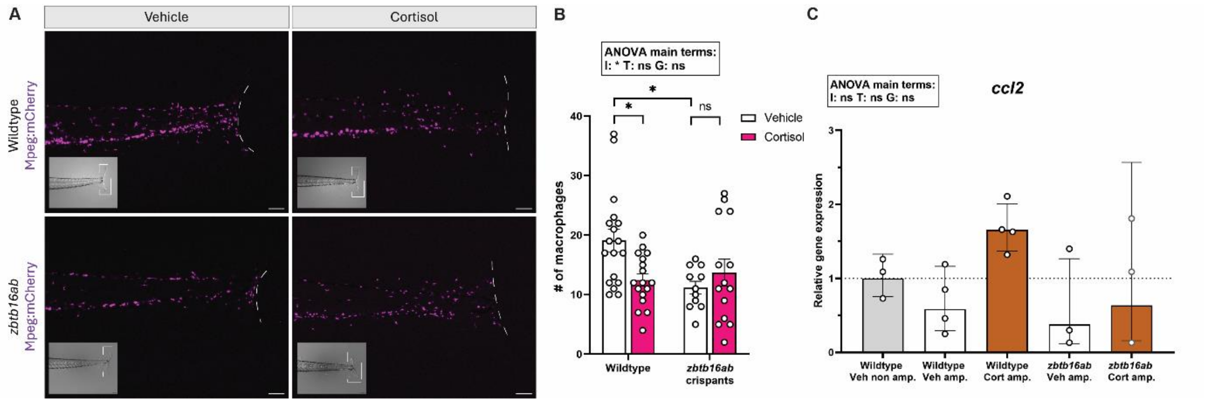
A
Vehicle
Cortisol
Wildtype
Mpeg:mCherry
zbtb16ab
Mpeg:mCherry
B
ANOVA main terms:
I: * T: ns G: ns
ns
Vehicle
Cortisol
# of macrophages
Wildtype
zbtb16ab crispants
C
ANOVA main terms:
I: ns T: ns G: ns
ccl2
Relative gene expression
Wildtype Veh non amp.
Wildtype Veh amp.
Wildtype Cort amp.
zbtb16ab Veh amp.
zbtb16ab Cort amp.

Aleksejeva, E, Houel, A, Briolat, V, Levraud, JP, Langevin, C & Boudinot, P 2016. Zebrafish plzf transcription factors enhance early type i ifn response induced by two non-enveloped rna viruses. *Dev Comp Immunol,* 57**,** 48-56.

Alsop, D & Vijayan, MM 2008. Development of the corticosteroid stress axis and receptor expression in zebrafish. *American Journal of Physiology-Regulatory, Integrative and Comparative Physiology,* 294**,** R711-R719.

Brinks, J, Van Dijk, EHC, Habeeb, M, Nikolaou, A, Tsonaka, R, Peters, HaB, Sips, HCM, Van De Merbel, AF, De Jong, EK, Notenboom, RGE, Kielbasa, SM, Van Der Maarel, SM, Quax, PHA, Meijer, OC & Boon, CJF 2018. The effect of corticosteroids on human choroidal endothelial cells: A model to study central serous chorioretinopathy. *Invest Ophthalmol Vis Sci,* 59**,** 5682-5692.

Brinks, J, Van Dijk, EHC, Kiełbasa, SM, Mei, H, Van Der Veen, I, Peters, HaB, Sips, HCM, Notenboom, RGE, Quax, PHA, Boon, CJF & Meijer, OC 2022. The cortisol response of male and female choroidal endothelial cells: Implications for central serous chorioretinopathy. *The Journal of Clinical Endocrinology & Metabolism,* 107**,** 512-524.

Chatzopoulou, A, Schoonheim, PJ, Torraca, V, Meijer, AH, Spaink, HP & Schaaf, MJM 2017. Functional analysis reveals no transcriptional role for the glucocorticoid receptor β-isoform in zebrafish. *Molecular and Cellular Endocrinology,* 447**,** 61-70.

Demichev, V, Messner, CB, Vernardis, SI, Lilley, KS & Ralser, M 2020. Dia-nn: Neural networks and interference correction enable deep proteome coverage in high throughput. *Nature Methods,* 17**,** 41-44.

Faught, E & Vijayan, MM 2019. Postnatal triglyceride accumulation is regulated by mineralocorticoid receptor activation under basal and stress conditions. *J Physiol,* 597**,** 4927-4941.

Gerault, M-A, Camoin, L & Granjeaud, S 2024. Diagui: A shiny application to process the output from dia-nn. *Bioinformatics Advances,* 4.

Koning, A, Habets, PC, Bogaards, M, Kroon, J, Van Santen, HM, De Bont, JM & Meijer, OC 2022. Mineralocorticoid receptor status in the human brain after dexamethasone treatment: A single case study. *Endocr Connect,* 11.

Schaaf, MJM, Champagne, D, Van Laanen, IHC, Van Wijk, DCWA, Meijer, AH, Meijer, OC, Spaink, HP & Richardson, MK 2008. Discovery of a functional glucocorticoid receptor β-isoform in zebrafish. *Endocrinology,* 149**,** 1591-1599.

Xie, Y, Tolmeijer, S, Oskam, JM, Tonkens, T, Meijer, AH & Schaaf, MJM 2019. Glucocorticoids inhibit macrophage differentiation towards a pro-inflammatory phenotype upon wounding without affecting their migration. *Dis Model Mech,* 12.
